# Molecular mechanism of dynein-dynactin complex assembly by LIS1

**DOI:** 10.1126/science.adk8544

**Published:** 2024-03-29

**Authors:** Kashish Singh, Clinton K. Lau, Giulia Manigrasso, José B. Gama, Reto Gassmann, Andrew P. Carter

**Affiliations:** 1 MRC Laboratory of Molecular Biology, Francis Crick Ave, Cambridge, CB2 0QH, UK; 2 Instituto de Investigação e Inovação em Saúde – i3S / Instituto de Biologia Molecular e Celular – IBMC, Universidade do Porto, 4200-135 Porto, Portugal

## Abstract

Cytoplasmic dynein is a microtubule motor vital for cellular organization and division. It functions as a ~4 MDa complex containing its cofactor dynactin and a cargo-specific coiled-coil adaptor. However, how dynein and dynactin recognize diverse adaptors, how they interact with each other during complex formation, and the role of critical regulators such as LIS1 remain unclear. Here, we determine the cryo-electron microscopy structure of dynein-dynactin on microtubules with LIS1 and the lysosomal adaptor JIP3. This structure reveals the molecular basis of interactions occurring during dynein activation. We show how JIP3 activates dynein despite its atypical architecture. Surprisingly, LIS1 binds dynactin’s p150 subunit, tethering it along the length of dynein. Together, our data suggest LIS1 and p150 constrain dynein-dynactin to ensure efficient complex formation.

Cytoplasmic dynein-1 (dynein) is an essential microtubule motor with roles in development, cellular function and disease (1, 2). It is unusual among cytoskeletal motors in requiring a large, 1.1 MDa cofactor called dynactin (3), which it binds in the presence of cargo-specific ‘activating adaptors’ (1, 4, 5). The formation of dynein-dynactin-adaptor (DDA) complexes is a key regulatory step in dynein activation (4–6). However, in the cell this process requires additional factors (7, 8), such as the key regulator LIS1 (9). This complexity raises two questions: how do diverse adaptors drive dynein-dynactin assembly, and how does LIS1 stimulate this process?

Activating adaptors share a long N-terminal coiled coil and motifs for interacting with specific parts of dynein or dynactin. Dynein is a dimer of heavy chains (DHCs), intermediate chains (DICs), light intermediate chains (DLICs) and light chains (10–13). All known activating adaptors contact the flexible C-terminal helix of DLIC (DLIC^helix^) via a variety of different motifs or domains (1, 14–16) (Fig. 1A). Additionally, some adaptors contain an HBS1 motif which binds DHC (17–19) and a Spindly motif which contacts dynactin (17, 19–21).

One putative family of adaptors that does not appear to conform with these rules includes the protein JIP3 (c-Jun N-terminal kinase-interacting protein 3) (1). This adaptor is important for trafficking neuronal lysosomes and autophagosomes (22–24) with dysfunctions leading to lysosome accumulations and neurological diseases (24–27). JIP3 binds DLIC^helix^ using its RILP Homology 1 (RH1) domain (28). However, the JIP3 N-terminal coiled coil, Leucine Zipper 1 (LZ1) (22), is half the length of other known dynein adaptors (Fig. 1A). Together with the absence of a defined HBS1 or Spindly motif, this difference make it unclear whether JIP3 activates dynein (1).

Along with the presence of an adaptor, DDA complex assembly also requires direct dynein-dynactin interactions. Dynactin features a short actin-related filament, a pointed end complex, and a shoulder domain with a long p150^Glued^ (p150) arm (20, 29–31). Dynein’s DHC tails bind the filament grooves (6) and its DIC N-terminus (DIC-N) interacts with p150 (32–37). The DIC-N:p150 interaction is critical for dynein function in cells (38) and formation of DDA complexes in vitro (39). However, as several studies suggest dynein and dynactin interact only weakly in the absence of an adaptor (4–6, 40), it is unclear when DIC-N binds p150 during DDA complex formation and why the interaction is critical for dynein’s activity.

The dynein regulator LIS1 was identified by mutations causing the brain developmental disorder Lissencephaly (41). It is important for dynein function in many eukaryotes (9, 40, 42–44), and recent evidence suggests it stimulates DDA complex formation (45–49). The current model is that LIS1 binds dynein’s motor domains (50–53) and opens up an autoinhibited form of dynein called the “phi-particle” (46, 48, 49, 54). However, LIS1 can still stimulate DDA formation in a dynein mutant where the phi-particle is constitutively open (45, 46). Furthermore, the phi-opening model does not explain other reported LIS1 functions, such as aiding recruitment of dynein-dynactin to microtubule plus-ends (40, 47, 55). In the absence of structural information of LIS1 in the context of the whole DDA complex, it is therefore not clear if the phi-opening model is sufficient to explain its different roles.

## JIP3 is an autoinhibited dynein activating adaptor

**Fig. 1:**
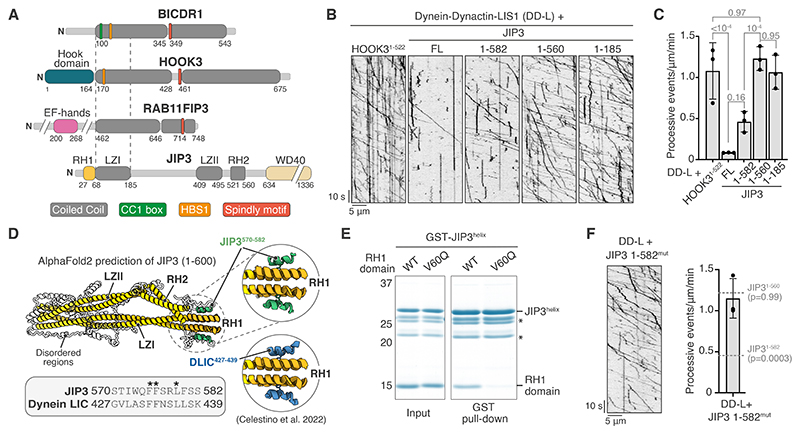
JIP3 is an autoinhibited activating adaptor for dynein motility in vitro. **(A)** Schematic representation of BICDR1 (also known as BICDL1), HOOK3, Rab11FIP3 and JIP3. The dotted lines illustrate the short coiled coil of JIP3 as compared to known dynein activating adaptors. **(B)** Kymographs of TMR-dynein-dynactin-LIS1 in the presence of HOOK3^1-522^, JIP3^FL^, JIP3^1-582^, JIP3^1-560^ and JIP3^1-185^. **(C)** Quantification of the number of processive events/μm microtubule/minute with the mean ± S.D. plotted. The total number of events analyzed were 1364 (HOOK3^1-522^), 95 (JIP3^FL^), 348 (JIP3^1-582^), 537 (JIP3^1-560^) and 513 (JIP3^1-185^). **(D)** An AlphaFold2 prediction of two copies of JIP3 (residues 1-600) showing an interaction between JIP3^helix^ (residues 570-582) and the RH1 domain. The JIP3^helix^ contains the same FFxxL motif as the DLIC^helix^ (residues 427-439 of the DLIC2 isoform) (bottom left), which has been shown to bind the RH1 domain (28) (bottom right). **(E)** Coomassie Blue-stained SDS-PAGE gel of purified recombinant protein mixtures prior to the addition of glutathione agarose resin (left) and of proteins eluted from glutathione agarose resin after GST pull-down (right). The RH1 domain construct was composed of JIP3^1-108^ and JIP3^helix^ construct was composed of GST-JIP3^563-586^. The asterisks denote the bands corresponding to degradation products. **(F)** Kymograph of TMR-dynein-dynactin-LIS1 in the presence of JIP3^1-582^mut (left). Quantification of the number of processive events/μm microtubule/minute with the mean ± S.D. plotted. The total number of events analyzed were 568. Statistical significance in comparison with JIP3^1-582^ and JIP3^1-560^ is depicted. The in vitro motility assays were performed with three technical replicates and statistical significance was determined using ANOVA with Tukey’s multiple comparison.

We initially set out to test if JIP3 can activate dynein-dynactin by quantifying its ability to stimulate processive movement using in vitro motility assays (5). We included LIS1 in all experiments to increase the robustness of dynein activation (45, 46). Under our assay conditions, the known activating adaptor construct HOOK3^1-522^ (4, 56) showed 1.07 ± 0.3 (SD) processive events/μm/min (Fig. 1B, C, Table S1). In contrast, full-length JIP3 (JIP3^FL^) displayed only a small number of processive events (Fig. 1B, C) which were not significantly higher than those observed in the absence of any adapter (Fig. S1A, Table S1). This behavior is reminiscent of other activating adaptors that are autoinhibited in their full-length form and can be activated by truncating their C-terminal segments (18, 57). We therefore generated JIP3 truncations and tested their ability to activate dynein. JIP3^FL^ contains an N-terminal RH1 domain, three coiled coils (LZI, LZII and RH2), followed by a WD40 domain (Fig. 1A). Removing the WD40 domain (JIP3^1-582^) did not significantly increase the number of processive events (Fig. 1B, C). In contrast, shorter constructs, JIP3^1-560^ and JIP3^1-185^, resulted in many long-distance dynein runs (Fig. 1B). The frequency of processive events (Fig. 1C, S1B), as well as the velocity and run lengths (Fig. S1C, D), were comparable to HOOK3^1-522^. The level of activation by JIP3 and the motile properties of resulting DDA complexes are thus similar to well-studied dynein adaptors like HOOK3.

The increase in dynein activation upon deletion of residues 561-582 suggests this region plays a role in autoinhibiting JIP3. An AlphaFold2 prediction of JIP3 (residues 1-600) shows a high confidence interaction between a helix within the 561-582 region (JIP3^helix^) and the RH1 domain (Fig. 1D, S1E) which resembles the interaction of the RH1 domain with the DLIC^helix^ (28) (Fig. 1D, S1E). To test the AlphaFold2 model, we performed pull-downs with purified proteins. We demonstrate that a JIP3^helix^ construct (GST-JIP3^563-585^) directly binds the RH1 domain (JIP3^1-108^) (Fig. 1E, S1F). This interaction was disrupted by an RH1-domain mutant (V60Q) (28) which is unable to bind DLIC^helix^ (Fig. 1E, S1F). Furthermore, the JIP3^helix^ contains the same FFxxL motif as the DLIC^helix^ (Fig. 1D) and mutating residues in this motif (JIP3^F576A^ or JIP3^L579A^) breaks the JIP3^helix^-RH1 interaction (Fig. S1F). Together these results show JIP3^helix^ binds to the RH1 domain in a similar manner to DLIC^helix^.

We next tested the effect of disrupting the JIP3^helix^-RH1 interaction on the ability of JIP3^1-582^ to activate dynein. Mutating the two phenylalanine residues to alanine in the JIP3 FFxxL motif (JIP3^1-582^mut) resulted in 1.15 ± 0.23 (SD) events/μm/min, which is comparable to that observed for JIP3^1-560^ and JIP3^1-185^ (Fig. 1F). Collectively these data show that JIP3 is autoinhibited by an intramolecular interaction between the JIP3^helix^ and the RH1 domain that likely hinders dynein’s DLIC^helix^ from binding.

### Structure of dynein-dynactin bound to JIP3 and LIS1

To understand how JIP3 recruits dynein-dynactin, we prepared complexes of dynein-dynactin with JIP3^1-185^ (DDJ^1-185^) or JIP3^1-560^ (DDJ^1-560^) and decorated them on microtubules in the presence of a non-hydrolysable nucleotide analogue, AMP-PNP. As in our in vitro motility assays, we included LIS1 to stimulate complex formation. We determined the DDJ structure with a cryo-EM processing pipeline involving microtubule signal subtraction (58) followed by focused 3D classification and 3D refinement (19). The cryo-EM datasets from the two different JIP3 constructs were later combined to better resolve regions present in both DDJ^1-185^ and DDJ^1-560^ structures (Fig. S2, Table S2).

**Fig. 2:**
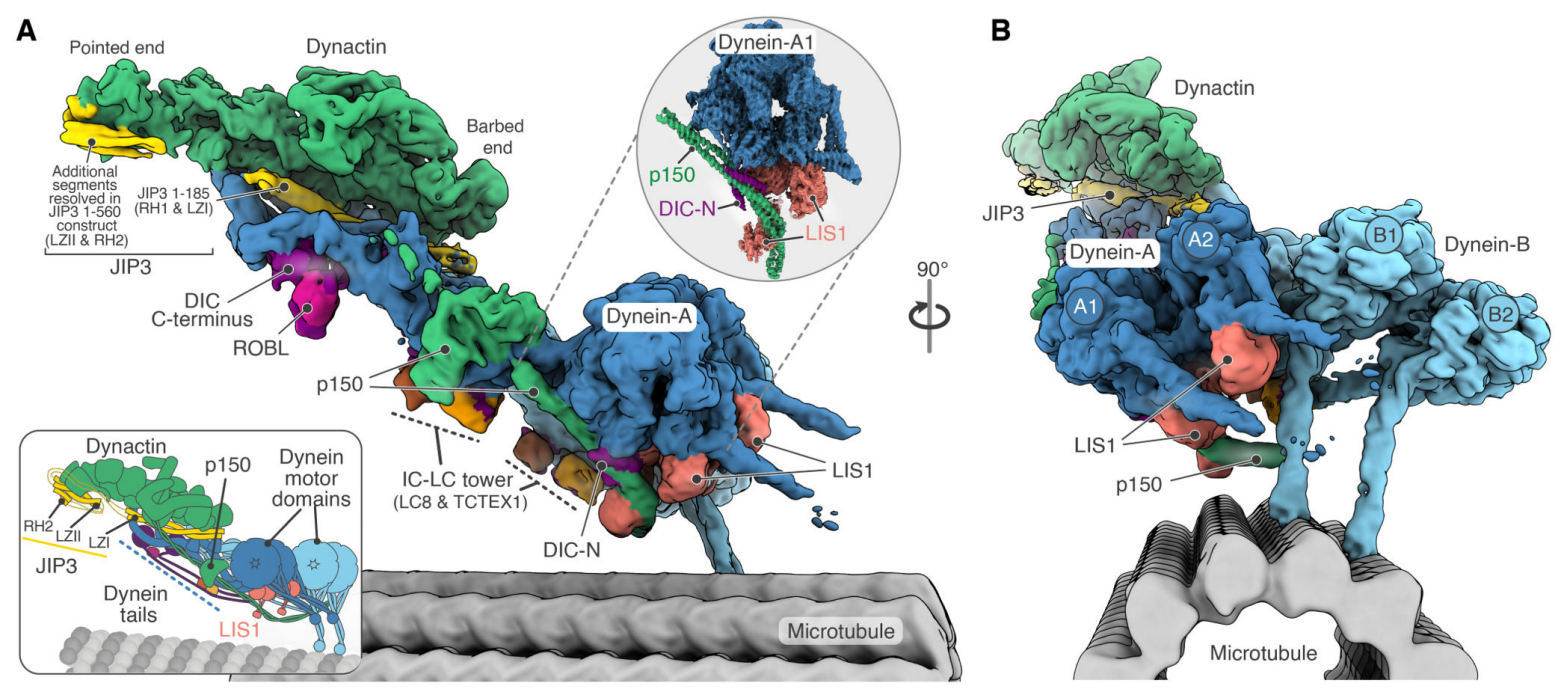
Cryo-EM structure of dynein-dynactin-JIP3-LIS1 on microtubules. **(A), (B)** Composite density map of dynein-dynactin bound to JIP3 and LIS1 overlaid on 13 protofilament microtubule. The zoom-in (grey circle) shows the locally refined map of dynein-A1 bound to p150, DIC-N and LIS1. Schematic representation of the complex is shown on the bottom left.

Our composite structure, generated from locally refined regions of the complex, shows that a single JIP3 adaptor recruits two dynein dimers per dynactin (Fig. 2A). When viewed with dynein walking towards the reader, dynein-A with its heavy chains A1 and A2 is located on the left whereas dynein-B is located on the right (Fig. 2B). The dynein-B motor domains are both in contact with the microtubule via their stalks, whereas the dynein-A motors are lifted away from the microtubule surface.

The structures contained ordered density for many functionally important parts that were too flexible to visualize in previous DDA structures (6, 19, 31, 56). These include much of dynactin’s p150 arm, structured segments of DIC bound to the dynein light chains LC8 and TCTEX1 (together referred to as the IC-LC tower) and the DIC-N of dynein-A. Of the few regions we cannot resolve, the most important are p150’s most N-terminal residues 1-390 and dynein-B DIC-N segments. Furthermore, we found two LIS1 dimers stably bound to the complex, one on each dynein-A motor domain. However, 3D classification shows no evidence of any DDA complexes with LIS1 bound to dynein-B (Fig. S2). To our surprise, one of the LIS1 dimers also contacts p150, thereby directly linking dynein to dynactin.

### Molecular basis of dynein-dynactin recruitment by JIP3

**Fig. 3:**
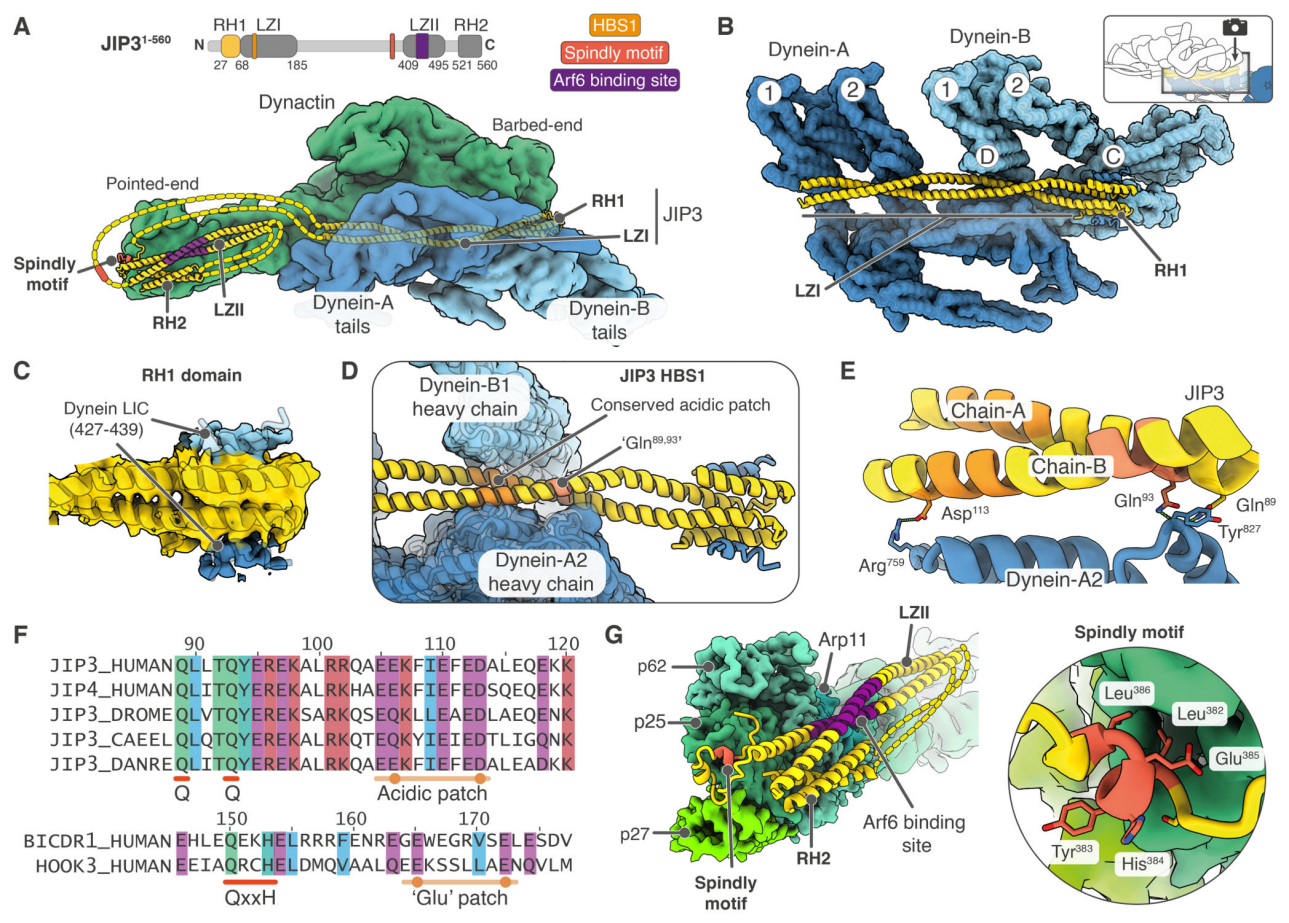
JIP3 interactions with dynein-dynactin. **(A)** Overall organization of JIP3 bound to dynein-dynactin. The unresolved regions which are predominantly unstructured are depicted as a dotted line. **(B)** Trajectory of JIP3 along the dynein heavy chain tails is shown using a top view of the complex. Dynactin segments are removed to aid visualization. **(C)** The N-terminal RH1 domain (yellow) has extra density on either side which is explained by the DLIC C-terminal helix (blue). The AlphaFold2 prediction of this interaction is displayed as cartoon inside the density. **(D)** JIP3 is sandwiched between heavy chains of dynein-B1 and dynein-A2. JIP3 contains an HBS1 which interacts with the dynein heavy chains using glutamine residues followed by an acidic patch. **(E)** JIP3 interaction with the dynein-A2 is shown. **(F)** Multiple sequence alignment of the JIP3 HBS1 from different organisms is shown. The residues involved in interactions with dynein are highly conserved. Sequences of HBS1 from BICDR1 and HOOK3 are shown for comparison. **(G)** JIP3 segments bound at the dynactin pointed end are shown. The JIP3 Spindly motif binds to the p25 subunit of dynactin whereas the LZII and RH2 foldback on each other and are bound along the p25 and p62 subunits (left). Spindly motif residues bound at the p25 subunit are shown on the right.

In our structure, JIP3 residues 24-185, containing the RH1 domain and ~18 nm long LZI coiled coil, bind along the cleft between the dynein tails and dynactin (Fig. 3A, B). This was resolved in both DDJ^1-185^ and DDJ^1-560^ structures (Fig S2). In addition, in the DDJ^1-560^ structure the C-terminal JIP3 residues 374-550, encompassing the LZII and RH2 coiled coils, bind at dynactin’s pointed end, with the intervening disordered residues 186-373 not visible. This arrangement differs from other structurally-characterized dynein adaptors, whose long coiled coils bind along the length of the dynactin filament extending all the way to the pointed end (19, 20, 56).

The interactions made by the RH1 domain and LZI are sufficient to promote dynein motility since the JIP3^1-185^ construct only contains those regions and can activate dynein in motility assays (Fig. 1B). The RH1 domain docks above the dynein-B1 tail (Fig. 3B) and shows extra density consistent with two copies of DLIC^helix^, one on each side (28) (Fig. 3C). Distance constraints make it likely that one DLIC^helix^ comes from dynein-A and the other from dynein-B. The LZI coiled coil is sandwiched between the dynein-A2 and B1 tails (Fig. 3B, D). Within the LZI, residues Gln^89^ and Gln^93^ interact with dynein-A2 via residue Tyr^827^. Additionally, a downstream patch of acidic residues in LZI interacts with both dynein-A2 (residues 759-830) and dynein-B1 (residues 420-460) (Fig. 3D, E). These JIP3-dynein interactions are analogous to the HBS1 motif (QxxY/H followed by a patch of glutamates) found in BICDR1 and HOOK adaptor families (17, 19) (Fig. 3F). However, the Gln^93^ of JIP3 sits on the opposite side of dynein Tyr^827^ compared to the equivalent Gln^150^ in BICDR1 (Fig. 3E, S3A). Furthermore, the downstream acidic patch is spaced differently with respect to the glutamine residue in these two adaptors (Fig. 3F). These differences in HBS1-dynein interactions are due to the different rotational orientation of the JIP3 coiled coil compared to BICDR1 (Fig. S3A) and illustrate the variability in how adaptors can recognize the DHCs.

With JIP3^1-560^, density corresponding to two coiled coils (~12 nm and ~6 nm long) docks against dynactin’s pointed-end subunits Arp11, p25, p27 and p62 (Fig. S3B). Based on the dimensions, we assigned the longer coiled coil to the LZII and the shorter one to the RH2 domain (Fig. 3G). Connected to LZII is a stretch of density containing a short alpha helix which docks onto p25 (Fig. 3G, S3B) at the site where the Spindly motif (LΦXEΦ, where Φ is hydrophobic) binds in other adaptor structures (19, 20). Upon searching the sequences flanking LZII, we found a JIP3 sequence, LYHEL (residues 382-386), that matches the Spindly motif consensus and explains our cryo-EM density (Fig. S3B). To support this assignment, we performed gel filtration assays to assess the interaction of JIP3 with a recombinantly-expressed pointed end complex (21). These showed that whereas JIP3^1-560^ binds the pointed end, mutations in the putative Spindly motif (L382A/Y383A/ E385A) disrupt this interaction (Fig. S3C). Together, our data show that JIP3 contains a previously unidentified Spindly motif that is critical for the JIP3-pointed end interaction.

LZII is important for JIP3 function due to its interaction with the small G-protein Arf6, which connects the adaptor to membrane cargos (22). However, the crystal structure of Arf6 bound to the JIP3 paralog, JIP4 (59), suggests Arf6 would sterically hinder LZII’s interaction with the pointed end (Fig. S3D). This discrepancy implies that either Arf6 prevents JIP3 binding to dynactin or Arf6 causes LZII to detach from the pointed end, but JIP3 remains attached via other means. To test these scenarios, we performed pull-downs using a JIP3 fragment (mouse JIP3^185-505^) containing the Spindly motif and LZII. We found this fragment can simultaneously bind both Arf6 and the recombinantly-expressed pointed end complex (Fig. S3E). A mutation in LZII (L439P, corresponding to a human disease mutation L444P (27)) disrupts Arf6 binding without affecting the pointed end interaction. Conversely, mutating the Spindly motif (L383A/E386A) disrupts the pointed end interaction without affecting Arf6 binding (Fig. S3E). We conclude that when JIP3 is on cargos, it binds the pointed end via its Spindly motif whereas the LZII binds Arf6 and is detached from dynactin (Fig. S3F).

### Dynactin’s p150 arm binds DIC-N, DHC and LIS1

**Fig. 4:**
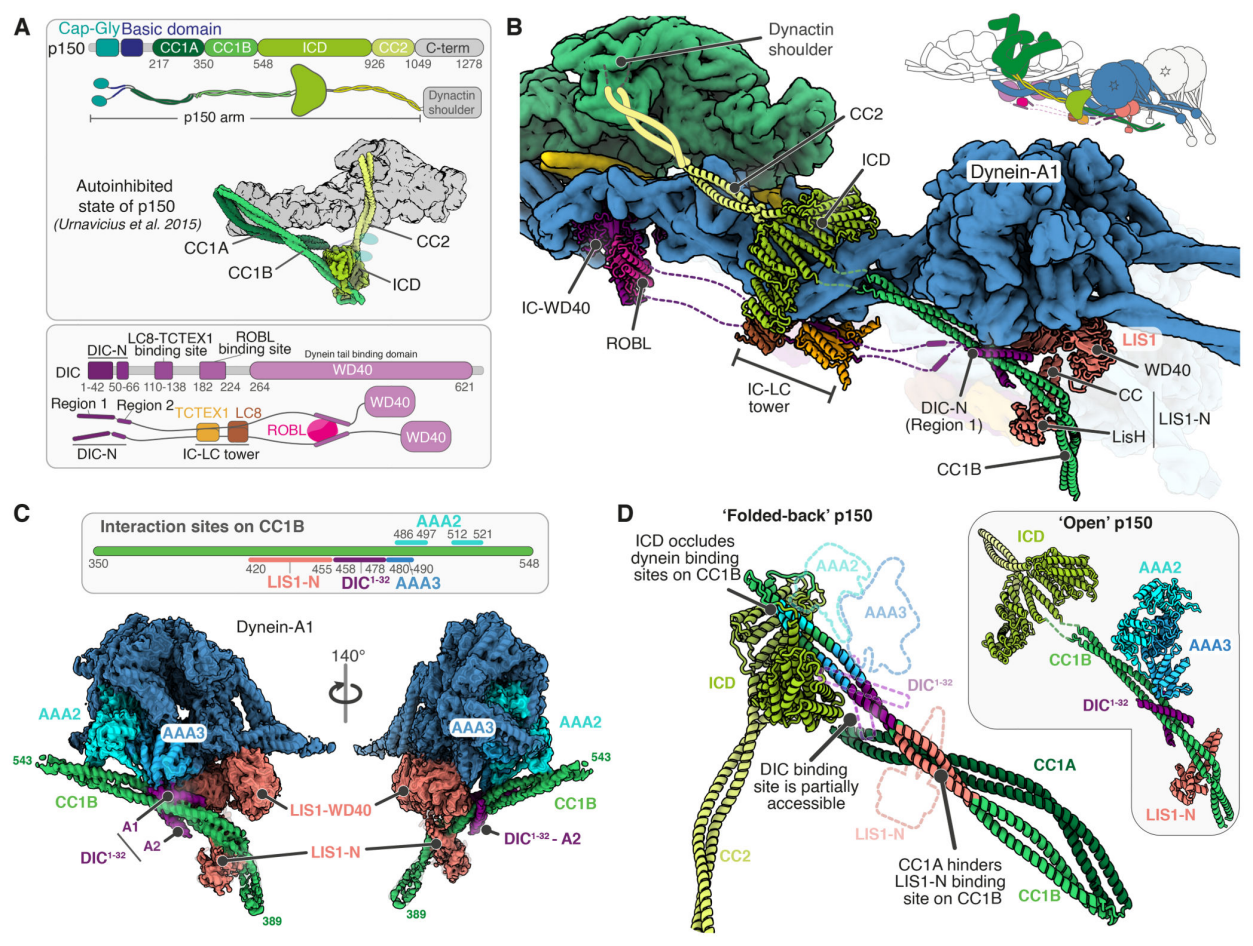
Organization of p150 and dynein intermediate chain. **(A)** Domain architecture of p150 (top) and its organization in the autoinhibited conformation is shown. Domain architecture of dynein intermediate chain and the interaction sites for dynein light chains are depicted (bottom). **(B)** Cartoon representation of p150 (green), dynein intermediate chain (magenta), dynein light chains (ROBL (pink), LC8 (brown) and TCTEX1 (golden)) and LIS1 (coral) bound to dynein-A1 (blue; depicted as surface). Unresolved segments are depicted as dotted or solid lines. **(C)** Schematic of interaction sites on p150-CC1B (top) and cryo-EM density of dynein-A1 motor domain bound by CC1B and LIS1 (bottom). CC1B is further bound by DIC-N region 1 (residues 1-32). **(D)** The regions of CC1B involved in binding the dynein motor domain, DIC^1-32^ and LIS1-N are mapped on the model of the autoinhibited p150. The open state of p150 solved in this study is shown on the right for comparison. The area covered by the two DIC^1-32^ helices, LIS1-N as well as the dynein AAA2 and AAA3 domains when bound to CC1B are shown using dotted lines.

The dynein-dynactin machinery contains two long flexible regions, the p150 arm and the DIC N-termini, both of which are important for complex assembly and function (32, 33, 39, 60–62) (Fig. 4A). The p150 arm extends as a coiled coil (CC2) from the dynactin shoulder (20, 29–31). It continues from C- to N-terminus as a globular intercoiled domain (ICD), followed by two coiled coils (CC1B and CC1A), a basic-rich region and CAP-Gly domains. The latter two sections mediate p150’s interaction with microtubules (20, 29–31). CC1B is the binding site for the very N-terminal ‘region 1’ helix of DIC-N (35, 36), although the exact interaction site is not known. Previous work showed CC1A and CC1B fold back on each other in isolated dynactin (29, 31, 63). This led to the idea that the folded-back CC1A/B hairpin is in an autoinhibited conformation that is opened upon DIC-N binding, although what role this plays in DDA assembly is also not clear.

In our JIP3- and LIS1-bound dynein-dynactin complex, p150 stably docks onto dynein allowing us to see much of its length. The ICD binds the dynein-A1 tail (residues 1160-1400) (Fig. 4B, S4A, S4B). The CC1A/B hairpin has opened up allowing CC1B to dock against the dynein-A1 motor (Fig. 4B). Although the N-terminal segments of p150 until CC1A are not visible, the interactions of CC1B with dynein positions them close to the microtubule (Fig. S9).

Our structure shows that CC1B not only binds DIC-N but acts as a major interaction hub for different parts of the complex (Fig. 4C). Its C-terminal end (residues 480-521) contains the binding site for the dynein-A1 motor. The dynein motor domain is built of a ring of six AAA domains (AAA1-AAA6) and here we see CC1B specifically contacts AAA2 and AAA3. The adjacent, N-terminal section of CC1B (residues 458-478) binds two alpha helices, one on each side of its coiled coil. We assign these helices to DIC-N based on previous reports (32, 35–37). Their length, together with an AlphaFold2 prediction of DIC-N bound to CC1B (Fig. S4C) suggests they correspond to the first part of DIC-N Region 1 (residues 1-32) (Fig. 4C). Furthermore, distance constraints suggest both DIC-N copies come from dynein-A.

Immediately N-terminal to the DIC-N binding site on CC1B is a section (residues 420-455) which binds LIS1 (Fig. 4B, C). LIS1 contains an N-terminal LisH domain and coiled-coil (together referred to as LIS1-N) required for dimerization (64) and a C-terminal WD40 domain which binds the dynein motor (52, 65, 66). The dynein-A1 bound LIS1 contacts CC1B via its LIS1-N domain. Notably, the CC1B coiled coil curves around LIS1-N and is redirected to run under the dynein motor domains (Fig. 4B, C). The interaction sites on CC1B/DIC-N/LIS1-N are highly conserved (Fig. S4D) and are also supported by an AlphaFold2 prediction of these segments (Fig. S4C).

### DIC-N opens dynactin’s p150 arm for LIS1 and dynein binding

The structure raises the question of which interactions are required to open up the CC1A/B hairpin. We used AlphaFold2 together with previously published cryo-EM maps and crosslinking data (20, 31) to generate a model of the autoinhibited form of p150 (Fig. 4D, S4E, S4F). We find that the ICD is docked onto CC1B in this model but is detached and connected to it by only a flexible linker in the open p150 (Fig. 4D). In the inhibited p150 the ICD blocks dynein motor binding to CC1B, showing an unanticipated role for the ICD in p150 autoinhibition. The model further shows that the previously identified hairpin, formed by CC1A binding to CC1B, is incompatible with LIS1-N binding. In contrast, the DIC-N binding sites are partially accessible. Comparison of the two p150 states suggests that DIC-N binding destabilizes the autoinhibited p150 as one DIC-N copy partially clashes with the ICD whereas the other would displace CC1A (Fig. 4D). This implies DIC-N binding opens up CC1A/B to allow CC1B to bind LIS1 and the dynein motor.

In support of the above model, we find LIS1 or LIS1-N are not pulled-down robustly by a CC1A/B construct (GST-CC1A/B), whereas they do bind CC1B (Fig. S5A). In contrast, a DIC-N construct (MBP-DIC-N) can bind CC1A/B, consistent with the partial accessibility of its binding site (Fig. S5B). This binding was sub-stoichiometric, which we hypothesized is due to the MBP-DIC-N construct being monomeric and hence making lower affinity interactions compared to a bivalent form found in dynein (32, 67). We thus changed the assay so that the beads are decorated with MBP-DIC-N instead of CC1A/B. The increased local concentration under these conditions allowed us to pull down CC1A/B in stoichiometric amounts. When DIC-N-decorated beads are incubated with CC1A/B and LIS1 or LIS1-N, we found a complex formed between all three components, providing evidence that DIC-N binding is required for LIS1 to interact with p150 (Fig. S5C). Together, our structure and pull-down assays suggest DIC-N binds p150 first and that the function of this interaction is to open up CC1A/B hairpin allowing it to bind the dynein motor and LIS1.

### The IC-LC tower promotes the CC1B/DIC-N interaction

Our cryo-EM structure shows two distinct globular densities below both dynein-A and B heavy chains (residues 1160-1230) (Fig. S6A). The resolution of our maps in this region was ~9 Å which is sufficient to identify them as belonging to the IC-LC tower based on its crystal structure (67, 68) (Fig. S6B). In each IC-LC tower, LC8 binds to the dynein heavy chain-1 (A1/B1) while TCTEX1 binds to the dynein heavy chain-2 (A2/B2) (Fig. S6A). Such a configuration suggests that the IC-LC tower plays a structural role. Firstly, the interaction with the DHCs reinforces the dynein tails lying parallel to one another. Secondly, the docking of the IC-LC tower positions DIC-N so it tethers the p150 close to the dynein-A motor domain.

### LIS1 stabilizes the pre-powerstroke state of dynein-A

**Fig. 5:**
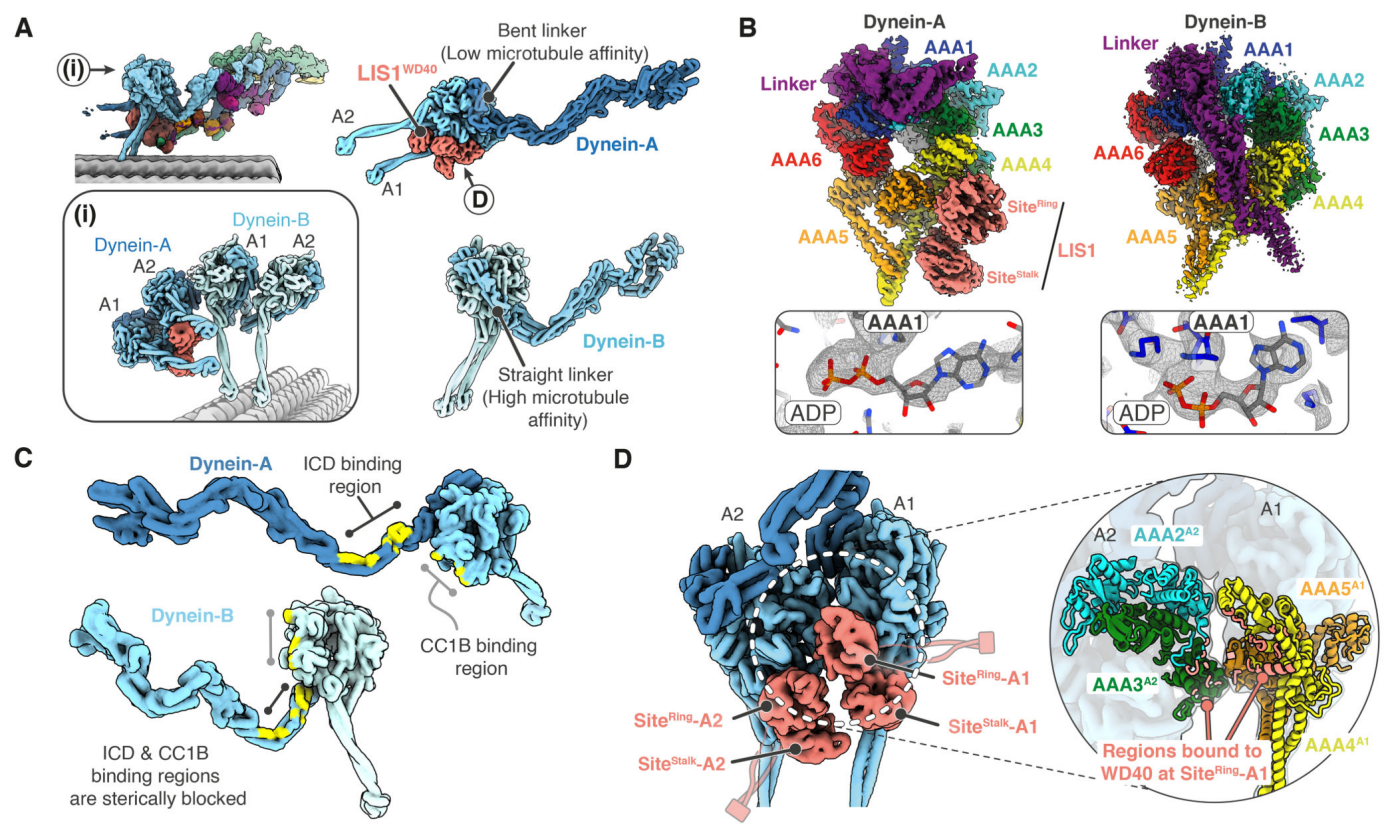
LIS1 stabilizes the pre-powerstroke state of dynein-A. **(A) (i)** Surface representation of LIS1 bound dynein-A and microtubule bound dynein-B. Dynein conformation and linker position is shown on the right. **(B)** Cryo-EM density of dynein-A and -B motor domains where the different subdomains and bound proteins are highlighted. **(C)** Regions involved in interacting with the ICD and CC1B segments of p150 are mapped (in yellow) onto the heavy chain of dynein-A (top) and dynein-B (bottom). **(D)** Surface representation of dynein-A motor domains with bound LIS1-WD40 domains (left). Interaction between dynein-A1 and A2 motor domains is shown (right) and the residues at the A1-A2 interface interacting with LIS1 are mapped (colored in coral).

In our structure, the two dynein dimers adopt different conformations (Fig. 5A). The LIS1-bound dynein-A motors have a bent linker docked onto their AAA2/AAA3 domains, indicating they are in a pre-powerstroke state (low microtubule affinity) (Fig. 5B). On the other hand, the microtubule-bound dynein-B motor domains have a straight linker docked onto AAA5 and are in a post-powerstroke state (high microtubule affinity) (Fig. 5B). In dynein-A motors we find the nucleotide density in both the AAA1 and AAA3 pockets is consistent with ADP (Fig. 5B, S6C). In contrast, the dynein-B motors contain ADP and AMP-PNP in AAA1 and AAA3, respectively (Fig. 5B, S6D). The nucleotide state of dynein-A is surprising since the presence of ADP in AAA1 typically corresponds to a high microtubule affinity state (53, 69), whereas dynein-A motors are detached from the microtubule in our structure. This implies LIS1 overrides the dynein conformation dictated by the bound nucleotides, stabilizing the pre-powerstroke state. Furthermore, the regions of dynein-A that bind to p150 are only accessible because its linker is bent and would be occluded by a straight linker (Fig. 5C). Thus, our data suggest LIS1 aids p150 docking via its ability to drive dynein-A into a pre-powerstroke conformation.

In our structure, the two LIS1 dimers contact the dynein-A motors via their WD40 domains. They bind at two sites (50, 52): one at the interface between AAA3-AAA4 (Site^ring^) and the other at the base of the stalk (Site^stalk^) (Fig. 5B). These are consistent with structures of LIS1 bound to isolated motor domains (50–53). In addition, in the context of our full dynein dimer, LIS1 sits in the cleft between the A1 and A2 motor domains, with the Site^ring^ WD40 domain on dynein-A1 contacting the AAA3 domain of dynein-A2 (Fig. 5D). This stabilizes dynein-A2 docking on AAA5 of dynein-A1 (Fig. 5D) and keeps the two motor domains parallel.

### LIS1 – p150 interaction is important for complex assembly

**Fig. 6:**
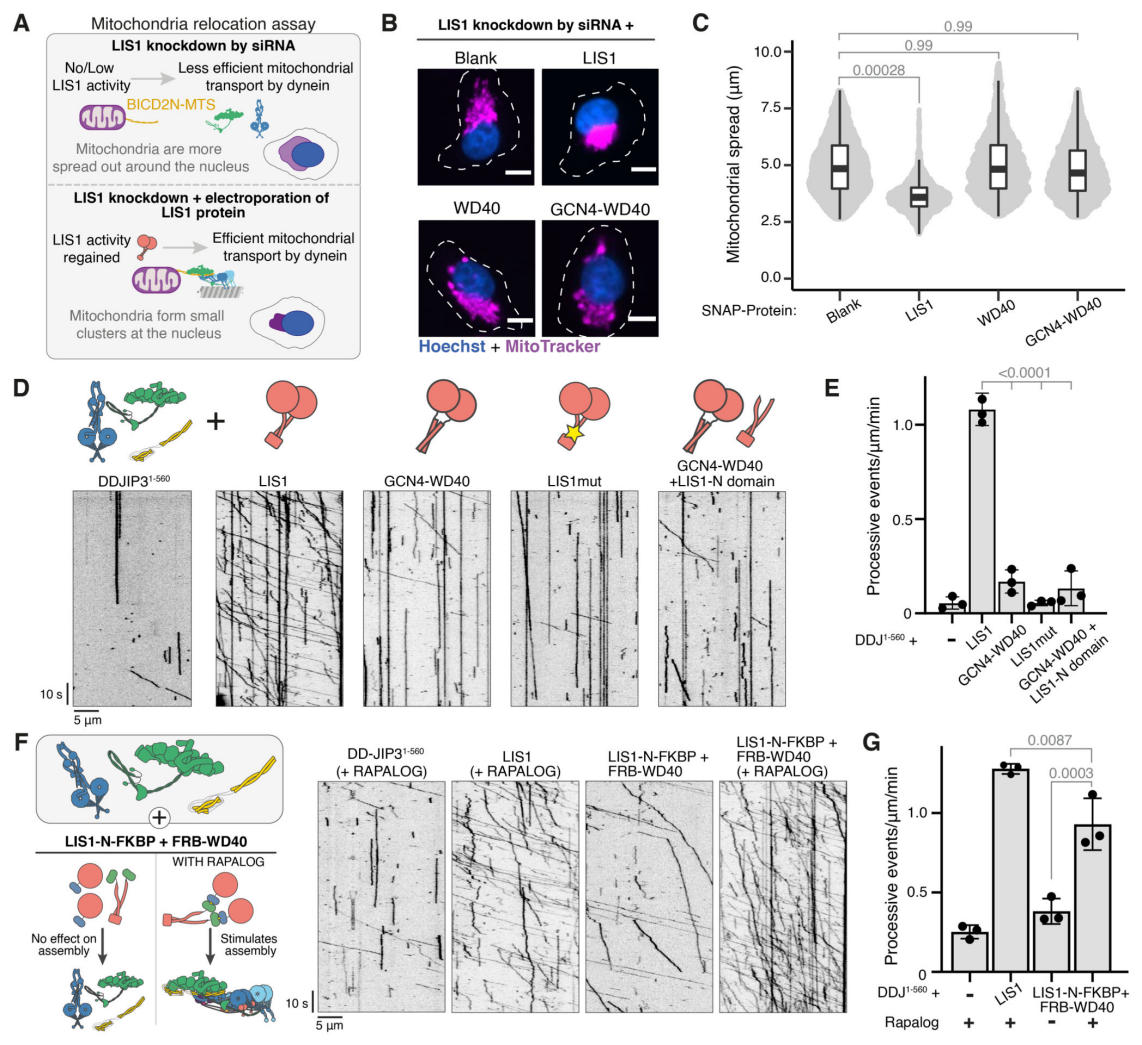
LIS1-N binding to p150 is important for dynein activation. **(A)** A schematic of the mitochondrial relocation assay is shown. **(B)** Representative images showing the distribution of mitochondria (magenta) in LIS1 siRNA-treated HeLa GFP-BICD2N-MTS cells electroporated with different SNAP-tagged LIS1 proteins. Scale bars represent 10 μm. **(C)** Quantification of mitochondrial spread (μm) in LIS1 knockdown HeLa GFP-BICD2N-MTS cells electroporated with different LIS1 proteins. Data are plotted from 3 biological replicates. The total cells quantified were 3922 (blank), 3830 (LIS1-SNAP), 2541 (WD40-SNAP) and 3655 (GCN4-WD40-SNAP). **(D)** Kymographs of TMR-dynein-dynactin-JIP3^1-560^ in the presence of (from left) blank, LIS1, GCN4-WD40, LIS1^mut^ and GCN4-WD40+LIS1-N. Cartoons depicting the LIS1 construct used are shown above each kymograph. **(E)** Quantification of the number of processive events per μm microtubule per minute with the mean ± S.D. plotted. The total number of movements analyzed were 74 (blank), 1592 (LIS1), 352 (GCN4-WD40), 116 (LIS1^mut^), and 225 (GCN4-WD40 + LIS1-N). Data are plotted from 3 technical replicates. **(F)** Schematic summarizing the effect on dynein assembly when LIS1-N and WD40 domains are present on the same molecule (left). Kymographs of TMR-dynein-dynactin-JIP3^1-560^ in the (from left) absence of LIS1, with LIS1, with FRB-WD40+LIS1-N-FKBP without and with rapalog are depicted (right). **(G)** Quantification of the number of processive events per μm microtubule per minute with the mean ± S.D. plotted. The total number of movements analyzed were 283 (blank), 675 (LIS1), 388 (FRB-WD40+LIS1-N-FKBP) and 665 (FRB-WD40+LIS1-N-FKBP with rapalog). Data are plotted from 3 technical replicates. Statistical significance was determined using ANOVA with Tukey’s multiple comparison.

The ability of LIS1 to stimulate DDA complex formation was attributed to its WD40 domains binding the dynein motor domains and disrupting the autoinhibited phi-particle (48, 49, 54). However, the interaction between LIS1-N and p150-CC1B raises the possibility that LIS1 has additional roles in dynein activation. To address this, we tested if the LIS1-WD40 domains alone are sufficient to activate dynein transport in cells. We used a mitochondria relocation assay, generating a Flp-In T-REX HeLa cell line expressing a GFP-tagged N-terminal-fragment of the activating adaptor BICD2 (BICD2N) fused to a mitochondrial targeting sequence (MTS) (Fig. 6A). In this cell line, mitochondria are clustered to the perinuclear region by BICD2N, and knockdown of LIS1 or dynein increases their spread (Fig. S7A, B). Importantly, LIS1 knockdown is rescued by electroporating the cells with LIS1 protein (Fig. 6B, C). The LIS1 used had a C-terminal SNAP tag (LIS1-SNAP) for detection but behaved identically to wild-type LIS1 (Fig. S7C).

To test whether the WD40 domain alone rescues LIS1 knockdown we used two constructs: one with a monomeric WD40 (WD40-SNAP) and a second dimeric construct where the LIS1-N dimerization domain was replaced by a GCN4 coiled coil (GCN4-WD40-SNAP) (Fig. S7D). Both constructs can bind to the dynein motor domain (Fig. S7E) but lack the ability to interact with p150-CC1B (Fig. S7F). The constructs were electroporated into LIS1 knockdown cells (Fig. S7G), but in contrast to wild-type LIS1, neither were able to rescue perinuclear mitochondrial clustering (Fig. 6B, C).

To dissect the role of the LIS1-N/CC1B interaction, we used our in vitro motility assay to check for assembly of active DDJ^1-560^ complexes. Consistent with our cellular observations, the presence of wild-type LIS1 led to multiple processive dynein runs whereas the monomeric WD40 or GCN4-WD40 constructs were unable to activate dynein (Fig. 6D, E, S7H). Additionally, we tested a construct in which the CC1B-interacting interface of LIS1 is mutated (Fig. S6F, I). This construct (LIS1^mut^) is a dimer and binds the dynein motor (Fig. S7D, E) but was unable to activate dynein transport (Fig. 6D, E), showing that binding to CC1B is important for LIS1 activity.

We found that LIS1-N alone was also unable to stimulate dynein activation, consistent with previous observations showing that the WD40-dynein interaction is necessary (45) (Fig. S7H). Furthermore, adding both LIS1-N and GCN4-WD40 constructs together did not increase the number of processive dynein runs (Fig. 6D, E). To determine if LIS1 activity requires a connection between LIS1-N and WD40, we used the rapalog-inducible FKBP-FRB system. LIS1-N was fused to FKBP and the WD40 domain to FRB. In the absence of rapalog, we observed similar numbers of processive dynein events as in the absence of LIS1 (Fig. 6F, G). Upon addition of rapalog, we observed a significant increase in dynein activation which resulted in ~73% as many processive events as observed for wild-type LIS1 (Fig. 6F, G). Together with our structure, this finding suggests that dynein activation requires LIS1 to simultaneously bind the dynein motor domain and the dynactin p150.

## Discussion

### Activation of the dynein machinery by JIP3

JIP3 activates long-range dynein-dynactin movement despite having a much shorter coiled coil than other cargo adaptors (1). This part of our work was previously released in our preprint (70) and is consistent with other contemporary studies (71, 72). In addition, here we identify a Spindly motif in JIP3. Recent studies concluded that many adaptors contain a disordered region between the end of their coiled coil and the Spindly motif (18, 19). JIP3 is an extreme example of this trend with a gap of 197 residues. Together these observations widen the criteria for identifying potential activating adaptors.

A JIP3 fragment containing just the RH1 domain and LZI coiled coil but lacking the Spindly motif is sufficient for activation. LZI spans the minimum distance required to interact with the tails of two dyneins providing an explanation for its length. We find, similarly, that short fragments of the adaptor BICD2 lacking a Spindly motif can activate dynein-dynactin (Fig. S8A-C). In our DDJ structure, there appear to be few, if any, interactions between the adaptor and the dynactin filament, something that was also observed in our previous high-resolution structure with the adaptor BICDR1 (19). Together these observations suggest that a minimal cargo adaptor requires only a coiled coil that can bind and orient the dynein tails so that they can interact with the grooves in dynactin’s filament. Although JIP3 and BICD2 do not absolutely require their Spindly motif in vitro, other adaptors including HOOK3 (73) and TRAK1/2 (74) do need it. We suspect these differences reflect the lack of sequence conservation among adaptors, suggesting some require the reinforcement from the Spindly-motif-pointed-end interaction to ensure their affinity for the dynein-dynactin complex. It remains an open question whether any activating adaptors lack the Spindly motif altogether.

Our data suggest LZII detaches from the pointed end upon cargo binding. When this happens, the distance from the pointed-end-bound Spindly motif to the Arf6 binding site on LZII is ~7.5 nm (Fig. S8D). This compares with ~46 nm between the Spindly motif and small-G-protein (Rab6) site on BICD2. Other adaptors show equivalent distances which lie between these two values. This means the dynein-dynactin-JIP3 complexes are positioned much closer to cargo membranes than some other DDA complexes. For organelles that could simultaneously recruit multiple types of adaptors this would allow more motors to attach the vesicle to the microtubule than would be possible if all adaptors are of the same length (Fig. S8E).

JIP3 is autoinhibited by an internal helix (JIP3^helix^) which binds to the RH1 domain in a similar manner as dynein’s DLIC^helix^. This helix-mimicry is distinct from the inhibition mechanisms of other activating adaptors. For example, in BICD2 (75, 76) and Spindly (18) a C-terminal coiled coil occludes the DLIC^helix^ binding site. JIP3 is part of a family of RH1 domain-containing proteins (77) including several potential dynein adaptors (JIP4 (72, 78), RILP (79), RILPL1 (80)) as well as the Myosin-Va interactor RILPL2 (81). Helical segments analogous to JIP3^helix^ are found in all members of this family suggesting the autoinhibition mechanism is conserved (Fig. S8F).

### Role of p150 and DIC-N in DDA assembly

Comparison of p150’s inhibited and open conformations (Fig. 4D) shows dynactin autoinhibition involves the previously identified CC1A/B hairpin (29, 31, 63) and an additional interaction involving p150’s ICD domain. Both CC1A and ICD need to detach for CC1B to bind dynein’s motor and LIS1. In addition, opening up p150 allows the ICD to contact dynein’s tail. Our observations implicate the ICD, which previously had no known function, as a key part of dynactin autoinhibition and DDA assembly.

Surprisingly, the DIC-N binding sites on CC1B are not fully occluded in the inhibited p150. Only the edges of the sites are partially blocked by the ICD and CC1A domains. This accessibility explains many pull-down studies showing an interaction between DIC-N and p150 (32–37, 39). Curiously, other studies using gel-filtration (5, 6), sucrose gradients (40) or single molecule assays (4) reported limited interaction of dynein and dynactin unless an activating adaptor is present. This may reflect the tendency of these methods to detect higher affinity interactions than pull-downs, suggesting that although dynein and dynactin can interact through DIC-N:p150, the formation of DDA further stabilizes this interaction. It was previously not clear why DDA complex formation requires DIC-N binding to CC1B (39). Here, we observe that LIS1 needs DIC-N present to bind p150 (Fig. S5). Together these results suggest that DIC-N is needed to open up p150 and allow subsequent interactions with LIS1 and the dynein motor.

Intriguingly, the interaction of DIC-N with p150 appears similar to its interaction with another dynein regulator Ndel1 (39). This explains the proposed competition between Ndel1 and p150 for dynein binding (37, 39, 82, 83) and suggests Ndel1 will need to be displaced before DIC-N can initiate DDA assembly (39, 82, 83).

### The IC-LC tower facilitates the p150 – DIC interaction during transport

The IC-LC towers are docked beneath the DHCs of both dynein-A and dynein-B. As the two differ in motor-conformation and binding to LIS1, it suggests neither factor affects the IC-LC interaction. Re-examination of our previous dynein-dynactin-BICDR1 (DDR) structure on microtubules (19), prepared without LIS1, showed evidence for light chains in a similar position. These observations suggest the standard location for dynein light chains is underneath DDA complexes making it unlikely they bind cargos, in contrast to some previous studies (84–86), but in agreement with others (68, 87).

In isolated dynein the IC-LC tower extends away from the motor (30) or docks onto one heavy chain via LC8 (6). In our structure the LC8 interaction is the same (6) whereas the additional TCTEX1 interaction is only possible because of the parallel arrangement of the DHC. This additional interaction could help stabilize the parallel arrangement of dynein motors needed for processive movement (6). Interestingly, light chain – heavy chain interactions are also found in dynein-2 (88) and axonemal dyneins (89–92). However, in all these cases the light chains make only a single contact with the relevant heavy chain, even when the heavy chains are parallel (89–91). This suggests the additional light chain – heavy chain interactions observed in our study are specific to DDA complexes.

The dynein-A IC-LC tower would constrain its DIC-Ns, and in turn p150, close to the dynein-A motor domains even in the absence of LIS1. This explains the extra density, similar in shape to p150, reported in our DDR structure (19). A recent single molecule study suggested that all DIC-Ns are sequestered during dynein-dynactin movement (39). This suggests dynein-A remains bound to CC1B and hence p150 remains tethered close to dynein-A during movement. The location of dynein-B DIC-Ns is not clear as they remain unresolved in our structure raising the question of how they are sequestered in the DDA complex. The IC-LC tower positioning, however, means they are constrained close to the dynein-B motor domain and the p150-CC1A.

There are several implications for positioning p150 close to dynein in moving DDA complexes. Firstly, it orients p150 such that the basic rich and CAP-Gly domains are held close to the microtubule (Fig. S9), consistent with their role in increasing dynein’s interaction with its track (60, 61, 93–95). Secondly, it means the p150 is available to interact with dynein-A1 whenever it is in its pre-powerstroke conformation. Continual binding/unbinding would be expected to interfere with dynein’s ATP hydrolysis cycle. This effect would be more consequential in single-dynein DDA complexes and may explain their slower stepping rate and velocity compared to two-dynein DDA complexes (56, 96).

### LIS1 keeps dynein in a low microtubule affinity state

A striking feature of our structure is that the LIS1 bound dynein-A motors are in a pre-powerstroke conformation with their stalks raised up and detached from the microtubule. In contrast our DDR structure (19), solved in the same AMP-PNP nucleotide condition but without LIS1, showed all motors in a post-powerstroke state bound to the microtubule. This suggests LIS1 stabilizes the pre-powerstroke conformation, overriding the conformation dictated by the nucleotide.

In our structure, dynein-B is in a post-powerstroke conformation and lacks LIS1 leading to the question of why it is different from dynein-A. Given reports that LIS1 encourages binding of a second dynein dimer (45, 46) it seems probable that dynein-B also engages LIS1 during DDA complex assembly. However, unlike the LIS1 molecules on dynein-A, which are additionally stabilized by the presence of p150, those on dynein-B are likely displaced by microtubule binding. This agrees with studies showing how LIS1’s interaction with dynein motors is incompatible with the microtubule-bound conformation (53) and implies dynein-B is the first motor to contact the microtubule.

These observations suggest our structure represents an intermediate on the pathway of DDA complex formation which is trapped after dynein-B has bound the microtubule, but before the complex starts moving. In the presence of ATP there are two likely outcomes: either dynein-A remains bound to LIS1/p150 and dynein-B alone starts moving or dynein-A loses LIS1, and both motors start walking. In our assays, in agreement with most (45, 46, 97) although not all (47) previous work, only ~22% of motile DDJ complexes move with LIS1 (Fig. S10A, B) and these move slower (Fig. S10C). This slow speed is consistent with single dynein motility (56). Therefore, LIS1 may remain attached to moving DDA complexes via dynein-A and p150 as we see in our structure, while movement is driven by dynein-B. The majority (75%) of DDJ complexes, however, lose LIS1 immediately upon movement initiation. This is shown by a single molecule assay in which DDJ complexes are bound to microtubules in the presence of LIS1 and AMP-PNP and movement is initiated by flowing in ATP (Fig. S10D-H). This suggests that the predominant route by which DDJ-LIS1 complexes start moving involves loss of LIS1 and the engagement of both dyneins with the microtubule.

### Interaction of LIS1 with p150 is important for DDA assembly

Recent evidence suggests a function of LIS1 is opening up the inhibited phi-particle by binding dynein motor domains (9, 48, 49, 54). However, we find that LIS1 constructs which bind dynein, but not p150, are unable to stimulate DDA complex formation in vitro or rescue loss of LIS1 in cells. This suggests that, although opening the phi-particle plays a role, it is not sufficient to promote DDA assembly. Instead, LIS1 binding p150 and dynein is additionally required.

Why might the LIS1 – p150 interaction be needed for the formation of active DDA complexes? Firstly, our structure suggests LIS1 reinforces the tethering of dynein to p150. After DIC-N opens CC1A/B, LIS1 binds CC1B. This favors robust DIC-N binding by reducing the chance that CC1A/B closes again. This would explain LIS1’s ability to enhance the recruitment of dynein to dynactin (or isolated p150) at microtubule plus ends in the absence of cargo adaptor (40, 47, 55).

Secondly, our structure provides evidence that LIS1 orients dynein-A for optimal dynactin and adaptor binding. The ability of LIS1 to contact both the CC1B and dynein allows p150 to dock along the heavy chain by binding the dynein tail and motor. We propose this tucks dynein under the p150 arm and holds its tail next to the dynactin filament. This would restrict the freedom of dynein and dynactin to prime them for efficient conversion into a motile complex in the presence of an activating adaptor.

### Model for formation of DDA complexes

Our data lead to a model of the molecular events during DDA complex assembly. In isolated dynactin, the p150 arm is autoinhibited by both a CC1A/B hairpin and interactions with the ICD. Dynein binding via DIC-N is required first to open up p150. Concurrently dynein’s phi-particle autoinhibition is relieved by LIS1 binding the dynein motor domain (46, 48, 49, 54). This likely makes the DHCs less constrained and is conducive for the subsequent steps in the assembly pathway. LIS1 then binds to the open p150, via LIS1-N, resulting in the formation of a primed complex containing interactions between p150, LIS1, the dynein motor and the dynein tail. We assume at this stage the cargo-bound activating adaptor is already bound to dynein via DLIC interactions, although, it is also possible dynein-dynactin-LIS1 complexes form without the adaptor present. In either case, the adaptor next moves into its final position, allowing the dynein tails to fully interact with dynactin. For complexes that contain two dyneins, the next step is recruitment of dynein-B, which our structure suggests is the first dynein to bind the microtubule. Finally, the predominant step is for LIS1 to dissociate from dynein-A as its motors engage the microtubule, allowing the DDA complex to move at full velocity.

LIS1 plays roles throughout the initiation of transport and in particular coordinates the interactions required for productive dynein-dynactin assembly. It is thus clear why it is essential for dynein function in cells.

## Materials and Methods

### Constructs

Full-length human JIP3 sequence (UniProt Q9UPT6-1) was codon optimized for expression in Sf9 cells (Twist Bioscience). Residues 1-1336 (JIP3^FL^), 1-582 (JIP3^1-582^), 1-560 (JIP3^1-560^) were subcloned with a N-terminal His6-ZZ tag followed by a TEV protease cleavage site (TEV) in a pAceBac1 vector. JIP3^1-582^_F576A/F577A and JIP3^1-560^_L382A/Y383A/E385A were generated using site directed mutagenesis of JIP3^1-582^ and JIP3^1-560^ constructs. Residues 1-560 were also inserted into a 2CT vector [N-terminal 6xHis::maltose binding protein (MBP) followed by a TEV protease cleavage site and C-terminal Strep-tag II (StTgII)] for bacterial expression. Constructs coding for JIP3 residues 1-108, 1-185 and 186-505 (28) were constructed by cloning fragments from mouse cDNA (isoform 3a; UniProt Q9ESN9-5) into the 2CT vector mentioned above. For the N-terminal JIP3 fragments, the only difference between the mouse and human sequence is residue 102 (lysine versus arginine, respectively). Mouse JIP3 186-505 differs from the equivalent human fragment at multiple residues, but the Spindly motif and the Arf6 binding site are identical. Constructs coding for BICD2 residues 2-201, 2-270 and 2-422 (14) were constructed by cloning fragments from human cDNA (UniProt ID: Q8TD16) into the 2CT vector mentioned above. Fragments coding for human JIP3^helix^ (563-585) and human Arf6 (12-175_Q67L) were inserted into a pGEX-6P-1 vector [N-terminal glutathione S-transferase (GST) followed by a Prescission protease (Psc) cleavage site and C-terminal 6xHis]. JIP3 mutants 1-108_V60Q (28), 186-505_L439P (28), 186-505_L383A/E386A, and 563-585_F576A/L579A were generated using site directed mutagenesis.

For the HOOK3 adaptor, pACEBac1-2xStrep-Psc-HOOK3^1–522^ was used and purified as previously described (56).

The dynactin pointed end complex construct (20) comprised of human ZZ-TEV-Arp11, p62 (isoform A, UniProt Q9UJW0-1), p25 and p27 in a pAceBac1 vector was used in the gel-filtration experiment (Fig. S3C). It was purified as described previously (20). For pulldown experiments, the pointed end complex was prepared as described (21) and was comprised of Arp11, p62, p27 and p25 tagged with a C-terminal 6xHis tag.

For dynein, full-length wild-type human cytoplasmic dynein-1 (5) and a “phi” mutant construct with mutations in the linker (R1567E and K1610E) to help overcome the autoinhibited conformation (6) were used. Dynein intermediate chain sequence 1-76 was cloned into the 2CT vector mentioned above. GST-Psc-DCTN1(residues 214-547)-GFP-6xHis, GST-Psc-DCTN1(residues 214-354)-GFP-6xHis and GST-Psc-DCTN1(residues 350-547)-GFP-6xHis were used for CC1, CC1A and CC1B fragment of DCTN1/p150glued (UniProt Q14203-1), respectively.

Full length human LIS1 with an N-terminal ZZ-TEV tag or a N-terminal ZZ-TEV tag plus a C-terminal SNAPf tag was cloned into a pFastbac vector (47). These constructs were used to generate LIS1 truncations: LIS1-N (residues 1-90), WD40 (residues 90-410) and GCN4-WD40 (GSGSVKQLEDKVEELLSKNAHLENEVARLKKLV + LIS1_81-410). For constructing LIS1^mut^ (LIS1_L3A_R6E_Q7A_K46E_K53E_K54E_T56A_S57A_I59A_R60E_Q62A_K63E_K64E_E6 9R), LIS1-N-FKBP (LIS1_1-84::5X-GS-linker::FKBP) and FRB-WD40 (FRB-T2098L::5X-GS-linker::LIS1_91-410), the sequences were first synthesized (Twist Bioscience) and then inserted into the pFastbac vector.

### Protein expression

For bacterial expression of proteins, SoluBL21 *E. coli* cells (Genlantis) were used and the expression was induced overnight at 18°C with 0.1-0.25 mM IPTG added at O.D of 0.6-0.8. Cells were harvested by centrifugation at 4,000 rcf for 20 min followed by a PBS wash and another centrifugation at 4,000 rcf for 20 min. The cell pellet was flash frozen in liquid nitrogen and stored at -80°C.

For expression in Sf9 cells, fresh bacmid DNA was transfected into Sf9 cells at 0.5x10^6^ cells/mL in 6-well cell culture plates using FuGENE HD (Promega) according to the manufacturer’s protocol (final concentration 10 μg/mL). After six days, 1 mL of the culture supernatant was added to 50 mL of 1x10^6^ cells/mL and cells were infected for five days in a shaking incubator at 27°C. The P2 virus was isolated by collecting the supernatant after centrifugation at 4,000 rcf for 15 min and stored at 4°C. For expression, 10 mL of P2 virus was used to infect 1 L of Sf9 cells at 1.5-2x10^6^ cells/mL for 72 hours in a shaking incubator at 27°C. Cells were harvested by centrifugation at 4,000 rcf for 10 min at 4°C, and washed with cold PBS. The cell pellet was flash frozen and stored at -80°C.

### Protein purification

Dynactin was purified from frozen porcine brains as previously described (31). Fresh brains were cleaned in homogenization buffer (35 mM PIPES pH 7.2, 5 mM MgSO4, 100 μM EGTA, 50 μM EDTA), and flash frozen in liquid nitrogen. Frozen brains were broken into pieces using a hammer. The brain pieces were blended and resuspended in homogenization buffer supplemented with 1.6 mM PMSF, 1 mM DTT, and 4 complete-EDTA protease-inhibitor tablets (Roche) per 500 mL. After thawing, the lysate was centrifuged in a JLA 16.250 rotor (Beckman Coulter) at 16,000 rpm for 15 min at 4°C. The supernatant was further clarified in a Type 45 Ti rotor (Beckman Coulter) at 45,000 rpm for 50 min at 4°C. After filtering the supernatant in a Glass Fibre filter (Sartorius) and a 0.45 μm filter (Elkay Labs), it was loaded on a column packed with 250 mL of SP-Sepharose (Cytiva) pre-equilibrated with SP buffer (35 mM PIPES pH 7.2, 5 mM MgSO4, 1 mM EGTA, 0.5 mM EDTA, 1 mM DTT, 0.1 mM ATP) using an Akta Pure system (Cytiva). The column was washed with SP buffer with 3 mM KCl before being eluted in a linear gradient up to 250 mM KCl over 3 column volumes. The peak around ~15 mS/cm was collected and filtered in a 0.22 μm filter (Elkay Labs) before being loaded on a MonoQ 16/10 column (Cytiva) pre-equilibrated with MonoQ buffer (35 mM PIPES pH 7.2, 5 mM MgSO4, 100 μM EGTA, 50 μM EDTA, 1 mM DTT). The column was washed with MonoQ buffer before being eluted in a linear gradient up to 150 mM KCl over 1 column volume, followed by another linear gradient up to 350 mM KCl over 10 column volumes. The peak around ~39 mS/cm was pooled and concentrated to ~3 mg/mL before being loaded on a TSKgel G4000SWXL column (Tosoh Bioscience) preequilibrated with GF150 buffer (25 mM HEPES pH 7.2, 150 mM KCl, 1 mM MgCl2) supplemented with 5 mM DTT and 0.1 mM ATP. The peak at ~114 mL was pooled and concentrated to ~3 mg/mL. 3 μL aliquots were flash frozen in liquid nitrogen and stored at -80°C.

For dynein purification, a cell pellet from 1 L expression was resuspended in 50 mL lysis buffer (50 mM HEPES pH 7.4, 100 mM NaCl, 10% (v/v) glycerol, 0.1 mM ATP) supplemented with 2 mM PMSF, 1 mM DTT, and 1 complete-EDTA protease-inhibitor tablet. Cells were lysed using a 40 mL dounce tissue grinder (Wheaton) with ~20 strokes. The lysate was clarified at 503,000 rcf for 45 min at 4°C using a Type 70 Ti Rotor (Beckman Coulter). The supernatant was incubated with 3 mL IgG Sepharose 6 Fast Flow beads (Cytiva) pre-equilibrated with lysis buffer for 4 hours at 4°C. The beads were then applied to a gravity flow column and washed with 150 mL of lysis buffer and 150 mL of TEV buffer (50 mM Tris-HCl pH 7.4, 150 mM KAc, 2 mM MgAc2, 1 mM EGTA, 10% (v/v) glycerol, 0.1 mM ATP, 1 mM DTT). For TMR labelled dynein, beads were transferred to a tube and incubated with 10 μM SNAP-Cell TMR-Star dye (New England Biolabs) for 1 hour at 4°C prior to the TEV buffer washing step. The beads were then transferred to a 5 mL centrifuge tube (Eppendorf) and filled up completely with TEV buffer. 400 μg TEV protease was added to the beads followed by overnight incubation at 4°C. The beads were transferred to a gravity flow column and the flow through containing the cleaved protein was collected. The protein was concentrated to ~2 mg/mL and loaded onto a TSKgel G4000SWXL column pre-equilibrated with GF150 buffer supplemented with 5 mM DTT and 0.1 mM ATP. Peak fractions were pooled and concentrated to ~2.5-3 mg/mL. Glycerol was added to a final concentration of 10% from an 80% stock made in GF150 buffer. 3 μL aliquots were flash frozen and stored at -80°C.

For purification of LIS1 constructs (full-length, truncations and mutants), a cell pellet from 1 L expression was resuspended in 50 mL lysis buffer B (50 mM Tris-HCl pH 8.0, 250 mM KAc, 2 mM MgAc2, 1 mM EGTA, 10% (v/v) glycerol, 0.1 mM ATP, 1 mM DTT) supplemented with 2 mM PMSF. Cells were lysed using a 40 mL dounce tissue grinder (Wheaton) with ~20 strokes. The lysate was clarified at 150,000 rcf for 30 min at 4°C using a Type 45 Ti Rotor (Beckman Coulter). The supernatant was incubated with 3 mL IgG Sepharose 6 Fast Flow beads (Cytiva) pre-equilibrated with lysis buffer B for 4 hours at 4°C. The beads were then applied to a gravity flow column and washed with 150 mL of lysis buffer B. For Alexa-647 labelled LIS1, beads were transferred to a tube and incubated with 10 μM SNAP-surface Alexa Fluor 647 dye (New England Biolabs) for 1 hour at 4°C followed by another washing step with lysis buffer B. The beads were then transferred to a 5 mL centrifuge tube (Eppendorf) and filled up completely with lysis buffer B. 400 μg TEV protease was added to the beads followed by overnight incubation at 4°C. The beads were transferred to a gravity flow column and the flow through containing the cleaved protein was collected. The protein was concentrated to ~3-5 mg/mL and loaded onto a Superdex 200 Increase 10/300 (Cytiva) column pre-equilibrated with GF150 buffer supplemented with 5 mM DTT and 10% glycerol. Peak fractions were pooled and concentrated to ~2-5 mg/mL. 5 μL aliquots were flash frozen and stored at -80°C.

For JIP3 constructs expressed in Sf9 cells, cell pellet from 1 L expression was resuspended in 50 mL lysis buffer C (50 mM HEPES pH 7.5, 100 mM NaCl, 10% (v/v) glycerol) supplemented with 2 mM PMSF, 1 mM DTT, and 1 complete-EDTA protease-inhibitor tablet. Cells were lysed using a 40 mL dounce tissue grinder (Wheaton) with ~20 strokes. The lysate was clarified at 150,000 rcf for 45 min at 4°C using a Type 45 Ti Rotor (Beckman Coulter). The supernatant was incubated with 3 mL IgG Sepharose 6 Fast Flow beads (Cytiva) pre-equilibrated with lysis buffer for 4 hours at 4°C. The beads were then applied to a gravity flow column and washed with 150 mL of lysis buffer C and 150 mL of TEV buffer B (50 mM Tris-HCl pH 7.4, 150 mM KAc, 2 mM MgAc2, 1 mM EGTA, 10% (v/v) glycerol, 0.1 mM ATP). The beads were then transferred to a 5 mL centrifuge tube (Eppendorf) and filled up completely with TEV buffer B. 400 μg TEV protease was added to the beads followed by overnight incubation at 4°C. The beads were transferred to a gravity flow column and the flow through containing the cleaved protein was collected. The protein was concentrated to ~2-4 mg/mL and loaded on a Superose 6 Increase 10/300 column (Cytiva) equilibrated with storage buffer (25 mM HEPES pH 7.5, 150 mM NaCl, 1 mM MgCl2, 1 mM DTT). Glycerol was added to a final concentration of 10% (v/v) and aliquots were flash frozen in liquid nitrogen and stored at -80°C.

For bacterially expressed MBP-StTgII tagged constructs, cell pellet from a 2 L culture was resuspended in 50 mL lysis buffer D (50 mM HEPES pH 8.0, 250 mM NaCl, 10 mM imidazole, 0.1% Tween 20, 1 mM DTT, 1 mM PMSF, 2 mM benzamidine-HCl) supplemented with 1 complete-EDTA protease-inhibitor tablet and 1 mg/mL Lysozyme. Cells were lysed by sonication and the lysate was clarified at 150,000 rcf for 30 min at 4°C using a Type 45 Ti Rotor (Beckman Coulter). The lysate was passed three times though 2 mL of pre-equilibrated Ni-NTA Agarose beads (Qiagen) in a gravity flow column and then washed with 300 mL of wash buffer A (25 mM HEPES pH 8.0, 250 mM NaCl, 25 mM imidazole, 0.1% Tween 20, 1 mM DTT, 1 mM PMSF, 2 mM benzamidine-HCl). Proteins were eluted with elution buffer A (50 mM HEPES pH 8.0, 150 mM NaCl, 250 mM imidazole, 1 mM DTT, 2 mM benzamidine-Cl). Fractions containing the protein were pooled, incubated overnight at 4°C with TEV protease and then incubated in batch with Strep-Tactin Sepharose resin (IBA) for 1 hour at 4°C, and washed with wash buffer B (25 mM HEPES pH 8.0, 250 mM NaCl, 0.1 % Tween 20, 1 mM DTT). Proteins were eluted on a gravity column with elution buffer B (50 mM HEPES pH 7.5, 150 mM NaCl, 3.5 mM desthiobiotin). The protein was further purified by size exclusion chromatography on a Superose 6 Increase 10/300 column (Cytiva) or Superdex 200 Increase 10/300 GL column (Cytiva) equilibrated with storage buffer (25 mM HEPES pH 7.5, 150 mM NaCl, 1 mM MgCl2, 1 mM DTT). Glycerol was added to a final concentration of 10% (v/v) and aliquots were flash frozen in liquid nitrogen and stored at -80°C.

For bacterially expressed GST-6xHis tagged constructs, cell pellet from a 2 L culture was resuspended in 50mL lysis buffer E (20 mM Tris-HCL pH 8.0, 300 mM NaCl, 15 mM imidazole, 10% glycerol, 1 mM DTT, 1 mM PMSF) supplemented with 1 complete-EDTA protease-inhibitor tablet and 1 mg/mL Lysozyme. Cells were lysed by sonication and the lysate was clarified at 150,000 rcf for 30 min at 4°C using a Type 45 Ti Rotor (Beckman Coulter). The lysate was passed three times through 2 mL of pre-equilibrated Ni-NTA Agarose beads (Qiagen) in a gravity flow column and then washed with 300 mL of lysis buffer D. Proteins were eluted with elution buffer C (20 mM Tris-HCL pH 8.0, 300 mM NaCl, 200 mM imidazole, 10% glycerol, 1 mM DTT). Fractions containing the protein were pooled and then incubated in batch with glutathione agarose resin (Thermo Fisher Scientific) for 1 hour at 4°C and washed with wash buffer C (20 mM Tris-HCL pH 8.0, 300 mM NaCl, 1 mM DTT). Proteins were eluted on a gravity column with elution buffer D (20 mM Tris-HCL pH 8.0, 300 mM NaCl, 20 mM reduced glutathione, 1 mM DTT). The protein was further purified by size exclusion chromatography on a Superose 6 Increase 10/300 column (Cytiva) equilibrated with GF150 (25 mM HEPES pH 7.5, 150 mM KCl, 1 mM MgCl2, 1 mM DTT). Glycerol was added to a final concentration of 10% (v/v) and aliquots were flash frozen in liquid nitrogen and stored at -80°C.

### Pull-down assays

For strep-tagged proteins, purified recombinant proteins (50 - 300 pmol each) were mixed in a total of 20 μL Strep-PD buffer (25 mM HEPES pH 7.5, 150 mM NaCl, 1 mM DTT) and incubated at room temperature for 30 min. For GST- and MBP-tagged proteins, GST-PD buffer (50 mM HEPES pH 7.5, 100 mM KCl, 1mM MgCl2, 1 mM DTT) was used. 4 μL were removed from the mixture (“input”) before adding 30 μL of a 50 % resin slurry (Glutathione Sepharose (Cytiva) for GST pull-downs, Strep-Tactin Sepharose (IBA Lifesciences) for Strep-tag II pull-downs and Amylose resin (NEB) for MBP pull-downs). The resin/protein mixture was rotated at room temperature for 30 min, and the resin was washed with 3 x 500 μL of the respective PD buffer, which for Strep-tag II pull-downs was supplemented with 0.05 % Tween-20. Proteins were eluted with 40 μL of 10 mM reduced L-glutathione in GST-PD buffer (glutathione agarose resin), 10 mM Maltose in GST-PD buffer (Amylose resin) or with 10 mM *d*-desthiobiotin in 100 mM Tris-HCL pH 8, 150 mM NaCl, 1 mM EDTA (Strep-Tactin Sepharose resin). 23 μL of PD buffer and 9 μL of 4x SDS-PAGE sample buffer were added to 4 μL of the input, and 9 μL of 4x SDS-PAGE sample buffer were added to 27 μL of the eluate before incubating samples at 95°C for 1 min. 5 μL of the input sample and 10 μL of the eluted sample were separated by SDS-PAGE and visualized by Coomassie Blue staining.

### Gel filtration for JIP3-dynactin pointed end binding

Dynein pointed end complex and JIP3^1-560^ or JIP3^1-560^ with mutations in the Spindly motif (L382A_Y383A_E385A) were mixed in 1:1 ratio in GF150 buffer with a final concentration of 10 μM in a volume of 60 μL. The mix was incubated for 30 min at 4°C and then 50 μL were loaded on a Superose 6 Increase 3.2/300 column (Cytiva) equilibrated with GF150.

### In vitro TIRF motility assays

Microtubules were made by mixing 1.5 μL of 2 mg/mL HiLyte Fluor 488 tubulin (Cytoskeleton), 2 μL of 2 mg/mL, biotinylated tubulin (Cytoskeleton) and 6.5 μL of 13 mg/mL unlabelled pig tubulin (5) in BRB80 buffer (80 mM PIPES pH 6.8, 1 mM MgCl2, 1 mM EGTA, 1 mM DTT). 10 μL of polymerization buffer (2× BRB80 buffer, 20% (v/v) DMSO, 2 mM Mg-GTP) was added followed by incubation at 4°C for 5 min. Microtubules were polymerized at 37°C for 1 h. The sample was diluted with 100 μL of MT buffer (BRB80 supplemented with 20 μM paclitaxel), then centrifuged on a benchtop centrifuge (Eppendorf) at 21,000 rcf for 9 minutes at room temperature. The resulting pellet was gently resuspended in 100 μL of MT buffer, then centrifuged again as above. 50 μL MT buffer was then added and the microtubule solution was protected from light. Microtubules were prepared the day before the assay was performed and stored in pellet form. Before usage, and every 5 hours during data collection, the microtubule solution was spun again at 21,000 rcf for 9 minutes, and the pellet resuspended in the equivalent amount of MT buffer.

Motility chambers were prepared by applying two strips of double-sided tape approximately 5 mm apart on a glass slide and then placing a coverslip on top. Before use, the coverslip were pretreated by sequentially sonicating in 3M KOH, water and then 100% ethanol followed by plasma treatment in Ar:O2 (3:1) gas mix for 3min. The coverslip was functionalized using PLL-PEG-Biotin (SuSOS AG), washed with 50 μL of TIRF buffer (30 mM HEPES pH 7.2, 5 mM MgSO4, 1 mM EGTA, 2 mM DTT), then incubated with streptavidin (1 mg/mL, New England Biolabs). The chamber was again washed with TIRF buffer, then incubated with 10 μL of a fresh dilution of microtubules (1.5 μL of microtubules diluted into 10 μL TIRF-Casein buffer (TIRF buffer supplemented with 50 mM KCl, 1 mg/mL casein and 5 μM paclitaxel)) for 1 min. Chambers were then blocked with 50 μL TIRF-Casein buffer.

Complexes were prepared by mixing each component in a total volume of 6 uL in GF150. The final concentrations in this mix were TMR-dynein at 0.1 μM, dynactin at 0.2 μM, adaptor at 2 μM and LIS1 at 6 μM. For LIS1 constructs where two components were added (for example: GCN4-WD40 + LIS1-N) each component was added at a final concentration of 6 μM in the initial mix. Complexes were incubated on ice for 15 min then diluted with TIRF-dilution buffer (TIRF buffer supplemented with 75 mM KCl and 1 mg/mL casein) to a final volume of 10 μL. 1 μL of this complex was added to a mixture of 15 μL of TIRF-Casein buffer supplemented with 1μL each of an oxygen scavenging system (4 mg/mL catalase, Merck; and 30 mg/mL glucose oxidase, Merck dissolved in TIRF buffer), 45% (w/v) glucose, 30% BME, 100 mM Mg-ATP and the final composition of this mixture was 25 mM HEPES pH 7.2, 4 mM MgSO4, 0.8 mM EGTA, 1.7 mM DTT, 45 mM KCl, 0.2 mg/mL catalase, 1.5 mg/mL glucose oxidase, 2.25% Glucose, 1.5% BME, 5 mM ATP, 3.75 μM paclitaxel, 3 nM TMR-dynein, 6 nM dynactin, 60 nM adaptor and 180 nM LIS1. This mix was flowed into the chamber. The sample was imaged immediately at 23°C using a TIRF microscope (Nikon Eclipse Ti inverted microscope equipped with a Nikon 100x TIRF oil immersion objective). For each sample, a microtubule image was acquired using a 488 nm laser. Following this a 500-frame movie was acquired (200 ms exposure, 4.1 fps) using a 561 nm laser. To analyze the data, ImageJ was used to generate kymographs from the tiff movie stacks. Events of similar length were picked to analyze velocity, run length and number of processive events/μm microtubule/min, using criteria outlined previously (5, 56). Velocity was calculated using pixel size of 105 nm and frame rate of 236 ms/frame. Three replicates were performed for each sample. Statistical significance was determined using ANOVA with Tukey’s multiple comparison test unless mentioned otherwise.

Our assay conditions have a higher ionic strength than we have used previously (56) where we diluted the initial DDA complex using buffer containing no KCl and the imaging buffer contained 25mM KCl. In line with previous observations (45) where higher ionic strength conditions have been shown to severely compromised the motility of dynein–dynactin–HOOK3 complexes in the absence of LIS1, we choose our present conditions in order to sensitize the assay to be able to best test the ability of different LIS1 constructs to activate DDA complexes.

For colocalization data, Alex647-labelled LIS1-SNAP construct (97% labelled) was used. The data was collected using a 561 nm and 640 nm laser along with a DV2 beam splitter (Photometrics), which projected each channel (TMR and Alexa Fluor 647) onto a different half of the image. Movie tif-stacks were split and kymographs were generated for each channel. Kymographs were overlaid using the ‘Colour:Merge’ function in ImageJ to generate a composite image. The kymographs were manually scored for processive events that showed colocalization.

For single-molecule ATP flow-in experiments, the motility chambers were prepared by using plexiglass slides with machine drilled holes 1 cm apart, into which inlet and outlet tubes (0.76 mm inner diameter, 10.5 cm in length, made of thermoplastic ethyl vinyl acetate) were attached using Araldite epoxy adhesive. On the other side, double-sided tape with a rectangular cutout (0.4 x 1.1 cm) was applied and the channel was sealed using a cleaned glass coverslip (as described above). The solutions were flowed in at a rate of 50 μL/min using a Infuse/Withdraw PHD 22/2000 Syringe Pump (Harvard Apparatus). The coverslip was functionalized using 30 μL of PLL-PEG-Biotin (SuSOS AG), washed with 80 μL of TIRF buffer and then 30 μL of streptavidin (1 mg/mL, New England Biolabs) was flowed in. The chamber was again washed with 60 μL TIRF buffer, then incubated with a fresh dilution of microtubules (1.5 μL of microtubules diluted into 40 μL TIRF-Casein buffer for 2 min. Chambers were then blocked with 60 μL TIRF-Casein buffer. DDJ^1-560^-L (with TMR-dynein and Alexa647-LIS1) were assembled and diluted to 10 μL as described above. From this protein mix, 0.5 μL was added to 40 μL of assay buffer (25 mM HEPES pH 7.2, 4 mM MgSO4, 0.8 mM EGTA, 1.7 mM DTT, 37.5 mM KCl, 0.2 mg/mL catalase, 1.5 mg/mL glucose oxidase, 2.25% Glucose, 1.5% BME, 2.5 mM AMP-PNP, 1.87 μM paclitaxel) and 30 μL of this buffer was flowed into the chamber. The chamber was then washed with 60 μL wash buffer (25 mM HEPES pH 7.2, 4 mM MgSO4, 0.8 mM EGTA, 1.7 mM DTT, 18.75 mM KCl, 0.2 mg/mL catalase, 1.5 mg/mL glucose oxidase, 2.25% Glucose, 1.5% BME, 1.87 μM paclitaxel). The motility was started by flowing in 30 μL of ATP Buffer (25 mM HEPES pH 7.2, 4 mM MgSO4, 0.8 mM EGTA, 1.7 mM DTT, 18.75 mM KCl, 0.2 mg/mL catalase, 1.5 mg/mL glucose oxidase, 2.25% Glucose, 1.5% BME, 5 mM ATP, 1.87 μM paclitaxel). The acquisition of a 300-frame movie (100 ms/frame exposure per channel with alternating channels) using 561 nm and 640 nm lasers was started at the same time point at which the flow of ATP buffer was initiated. The kymographs were manually scored for processive events and colocalization. As controls, we looked at background binding between LIS1 and dynein in the absence of dynactin and JIP3. For this we prepared the complex and substituted dynactin and adaptor with GF150 buffer. In addition, to quantify the amount of LIS1 dissociation due to assay conditions (including effects of fluid flow) we also collected data where buffer containing AMP-PNP was flowed in instead of ATP containing buffer.

### Cryo-EM sample preparation

To polymerize microtubules, tubulin was diluted in microtubule buffer (25 mM MES pH 6.5, 70 mM NaCl, 1 mM MgCl2, 1 mM EGTA, 1 mM DTT) with 6 mM GTP (MilliporeSigma) such that the final concentration of tubulin was 5 mg/mL (45 μM) tubulin and that of GTP was 3 mM. The mixture was incubated on ice for 5 min, and microtubules were polymerized at 37°C for ~1.5 hours. To stabilize the microtubules, microtubule buffer was supplemented with 20 μM paclitaxel. The microtubules were pelleted on a benchtop centrifuge (Eppendorf) at 20,000 rcf for 8 min at room temperature. The supernatant was discarded and the pellet was resuspended in microtubule buffer with paclitaxel by pipetting up and down with a cut tip. The microtubules were pelleted and resuspended again using an uncut tip. The concentration was estimated using Bradford reagent (BioRad) and diluted to ~0.65 mg/mL (~6 μM). To assemble the dynein-dynactin-JIP3 complex in the presence of LIS1, the purified proteins were mixed in a 1:2:60/44:32 molar ratio (0.27 μM dynein, 0.54 μM dynactin, 16 μM JIP3^1-185^ or 12 μM for JIP3^1-560^ and 8.7 μM LIS1) in GF150 buffer supplemented with 1 mM DTT in a total volume of 10 μL and incubated on ice for 30 min. To bind the complex to microtubules, 9 μL complex was mixed at room temperature with 5 μL microtubules and 9 μL binding buffer A (77 mM HEPES pH 7.2, 51 mM KCl, 13 mM MgSO4, 2.6 mM EGTA, 2.6 mM DTT, 7.68 mM AMP-PNP, 13 μM paclitaxel). After 15 min, the complex-bound microtubules were pelleted at 20,000 rcf for 8 min at room temperature. The pellet was resuspended in binding buffer B (30 mM HEPES pH 7.2, 50 mM KCl, 5 mM MgSO4, 1 mM EGTA, 1 mM DTT, 3 mM AMP-PNP, 5 μM paclitaxel, and 0.01% IGEPAL (MilliporeSigma) using an uncut tip. 3.5 μL was applied to freshly glow discharged Quantifoil R2/2 300-square-mesh gold grids (Quantifoil) in a Vitrobot IV (ThermoFisher) at 100% humidity and 20°C, incubated for 30 s, and blotted for 0.5-2 s before being plunged into liquid ethane.

### Cryo-EM data collection and image processing

The samples were imaged using a FEI Titan Krios (300 kV) equipped with a K3 detector and energy filter (20 eV slit size) (Gatan) using automated data collection (ThermoFisher EPU). Movies were acquired at 81,000 X magnification (1.06 Å/pixel, 100 μm objective aperture, ~2.2 sec exposure, 54 frames, fluence of ~54 e-/Å^2^ and -1.2 to -3 μm defocus range).

Global motion correction and dose-weighting were performed in Relion-4.0 (99) using MotionCor2 (100) with a B-factor of 150 and 5X5 patches. The power spectrum of aligned frames was used for CTF estimation by CTFFIND4 (101). Microtubules were picked from the micrographs using the single particle picking mode in crYOLO (102) with a model that was trained on manually picked micrographs (~100) in filament mode. The output coordinates along each microtubule were spaced by 81 Å. The coordinates were then resampled to a 4 nm interval using the multi-curve fitting script described previously (58) (https://github.com/PengxinChai). The coordinates for each microtubule were then further split into segments of 10-17 coordinates as this gave better results with the following microtubule subtraction step. Finally, microtubule subtraction was performed as described previously (58). The subtracted microtubules were then used to pick dynein-dynactin-JIP3 complexes using crYOLO (103). The picked particles were imported into CryoSPARC (104) followed by 2D classification. The particles in classes showing dynein-dynactin were used for ab-initio initial model generation. Three initial models were generated which were then used for a round of heterogeneous refinement using all particles. Particles belonging to the 3D class which displayed good density for dynein-dynactin were used for a round of non-uniform refinement and then imported into Relion-4.0 for particle polishing and local refinements. The consensus structures of complexes with JIP3^1-185^ and JIP3^1-560^ appeared identical except the JIP3 densities at the pointed end which were only present in the DDLJ^1-560^ dataset. Therefore, the two datasets were merged at this stage. The merged data was used for local refinements and classifications for all parts of the complex except the dynactin pointed end which was solved using only the DDLJ^1-560^ dataset. The processing pipeline including mask used, particle numbers, resolution of maps, B-factors and classification parameters can be found in Fig. S2. Data collection and image processing statistics can be found in Table S2.

### Model building and refinement

Model building and restrained flexible fitting of models in the density was done in COOT (105) and refinements were performed in PHENIX (106). Models from the Protein Data Bank (PDB) or AlphaFold2 predictions (see section below) were docked into the respective cryo-EM maps as a rigid body using UCSF Chimera (107). All refinement statistics can be found in Table S2.

AlphaFold2 predictions of JIP3 (1-185) and the JIP3 RH1 domain bound to two DLIC^helix^ were used as starting models for JIP3 (residues 24-185) and DLIC^helix^ (residues 429-441). The registry of the JIP3 coiled-coil was determined using the characteristic shape of the RH1 domain and side chain resolution at the HBS1 site. One DLIC^helix^ was assigned to dynein-A whereas the other to dynein-B based on distance constraints. However, it is possible that both DLIC^helix^ could be contributed by the same dynein molecule. Further, we were unable to resolve the flexible and disordered loop connecting the DLIC^helix^ to the N-terminal RAS-like DLIC domain and two out of the four DLIC^helix^ copies.

Dynein tails (residues 20-1444) with bound dynein subunits (DLIC RAS-like domain; residues 37-373, C-terminal regions of DIC; residues 182-206 and 217-595, ROBL; residues 3-95) were built using PDB-7Z8F (19) as the starting model. The dynactin filament was built based on PDB-7Z8F and PDB-6ZNL (20).

The JIP3 Spindly motif and its flanking segments (residues 374-405) interacting with the dynactin pointed end complex were built manually guided by the density and the AlphaFold2 prediction of the Spindly motif bound to p25 and p27 dynactin subunits. The longer coiled-coil at the pointed end was assigned to JIP3 LZII (residues 406-495) as we observed fragmented density connecting the Spindly motif to this coiled-coil. Additionally, LZII is the only sequence in JIP3 that can form a coiled-coil of sufficient length to match our density. The lower short coiled-coil at the pointed end had a similar length as the JIP3 RH2 domain (residues 518-551 were built) and was therefore assigned to this sequence. The orientation of the RH2 domain was determined based on LZII. AlphaFold2 prediction of JIP3 1-600 was used to obtain the starting models for the LZII and RH2 domains. The disordered loop connecting the JIP3 1-185 segment to the Spindly motif as well as that between the LZII and RH2 coiled coils were flexible and not visualized in our maps.

The IC-LC tower (LC8, TCTEX and DIC residues 110-140) bound to DHC helical bundles 8/9 was modelled using a combination of the PDB-7Z8F and the AlphaFold2 prediction of IC-LC tower bound to a single DHC. The AlphaFold2 model predicted the interaction of LC8 with DHC tail-1 as can be seen from the fit in the experimental density (Fig. S6B). DHC tail-2 was built taking PDB-7Z8F as the starting point followed by flexible fitting in COOT. The disordered DIC loops connecting the IC-LC tower to the DIC C-terminal segments (from residue 182) and the DIC^1-32^ helix were too flexible and not visualized in our maps.

The p150 ICD and CC2 (residues 557-987) were built using an AlphaFold2 model of these segments. The p150 CC1B-DIC-LIS1-N AlphaFold2 prediction was used to model CC1B (residues 391-543), DIC^1-32^ and LIS1-N (residues 1-80). The predicted model was docked in the experimental density based on the DIC^1-32^, LIS1-N and CC1B. The orientation of the DIC^1-32^ helices and LIS1-N along with the spacing between these two binding sites on CC1B were identical in both the AlphaFold2 model and the cryo-EM density. This also aided in identifying the registry of the CC1B fragment. The built registry of the CC1B coiled-coil was further confirmed based on hydrophobic residues 513-520, which could be distinguish at the resolution of ~4.2 Å. CC1B bends around the LIS1-N, which was built using flexible fitting in COOT followed by refinement in PHENIX. We assign both the DIC^1-32^ helices to the dynein-A as they are more likely to bind p150-CC1B due to proximity. Our structure suggests only one of the two DIC^1-32^ helices from dynein-B could theoretically reach CC1B. However, for this interaction to occur, the rest of the DIC N-terminus of dynein-B would need to adopt an extremely stretched conformation, which we consider unlikely. The N-terminal segments of p150 consisting the CAP-GLY domains and CC1A coiled coil (residues 1-390) along with the loop connecting CC1B to ICD (residues 544-556) and the C-terminal half of CC2 coiled coil (residues 988 to 1088) were too flexible and not visualized in our maps.

Dynein-A motor domain (residues 1444-4646) was built using PDB-5NUG (6). The cryo-EM density of dynein-A was consistent with ADP in AAA1, ATP in AAA2 and ADP in AAA3. Nucleotide density in AAA4 was fragmented but correlated well with an ADP which is in line with other structures of the dynein motor domain in pre-powerstroke state (PDB-4RH7 (108) and PDB-8FDT (53)). Dynein-B motor domain (residues 1327-4646) was built using PDB-7Z8G (19) which was solved under similar buffer conditions. Cryo-EM density of dynein-B was consistent with ADP in AAA1, ATP in AAA2 and AMP-PNP in AAA3 and AAA4 in line with the previous structure.

For generating the composite map, the locally refined maps (Fig. S2) were placed into the consensus map of dynein-dynactin-JIP3^1-560^-LIS1 filtered to 20 Å using UCSF Chimera (107). The maps were then aligned with respect to each other using overlapping regions using the ‘fit-in-map’ function. The maps were then merged using the ‘volume max’ function and then filtered to 15 Å. Although the raw data show that there is flexibility in the orientation of the complex relative to the MT (Fig. S2), for illustrative purposes the composite map was overlaid onto the 13 protofilament microtubule (from (19)) by matching the configuration of the microtubule binding domains of dynein-B with the tubulin dimers with PDB-6RZB (109).

### AlphaFold2 prediction

All structure predictions were performed using AlphaFold2 through a local installation of ColabFold (110) running MMseqs2 (111) for homology searches and AlphaFold2 (112) or AlphaFold2-Multimer (113) for the predictions of single or multiple chains, respectively. JIP3 (UniProt Q9UPT6) regions 1-185 and JIP3 1-600 were predicted by running ColabFold 1.2.0 on two copies of these segments. The RH1 domain-DLIC^helix^ interaction was predicted by running ColabFold 1.2.0 on two copies each of JIP3 1-99 and DLIC2 427-439 (UniProt O43237). IC-LC tower was predicted by running ColabFold 1.3.0 using two copies each of DIC (100-150) (UniProt Q13409), Tctex1 (UniProt P63172) and LC8 (UniProt P63167). Interaction of IC-LC tower with dynein heavy chain was predicted by running ColabFold 1.3.0 using two copies each of DIC (100-150), TCTEX1, LC8 and a single copy of dynein heavy chain (1110-1350) (UniProt Q14204). Dynactin p150 (UniProt Q14203-1) regions were predicted by running ColabFold 1.3.0 using two copies of DCTN1 (1-556) for Cap-Gly domain to the CC1B segment and of DCTN1 (557-987) for the ICD-CC2 segment. LIS1 (UniProt P43034) was predicted by running ColabFold 1.3.0 using two copies of this protein. Interaction between CC1B-DIC-N-LIS1-N was predicted by running ColabFold 1.3.0 on two copies each of DCTN1 (350-547), DIC (1-34) and LIS1 (1-84). The adaptors JIP3, HOOK3 (UniProt Q86VS8), BICDR1 (UniProt Q6ZP65), BICD2 (UniProt Q8TD16), RILP (UniProt Q96NA2), RILPL1 (UniProt Q5EBL4) and RILPL2 (UniProt Q969X0) were predicted by running ColabFold 1.2.0 on two copies of each protein. Interactions of RH1 domains with C-terminal helical elements in RILP, RILPL1 and RILPL2 were predicted by running ColabFold 1.3.0 using two copies of residues 21-103 and 337-353 for RILP, residues 1-141 and 353-367 for RILPL1 and residues 25-106 and 185-211 for RILPL2. The predicted aligned error (PAE) with respect to residue was mapped onto the predicted structure using PointPAE 1.0 (https://github.com/sami-chaaban/PointPAE) (doi: 10.5281/zenodo.6792801), and ChimeraX (114) was used for visualization.

### Size-exclusion chromatography-multi angle light scattering (SEC-MALS)

SEC-MALS measurements were performed using a Wyatt Heleos II 18 angle light scattering instrument coupled to a Wyatt Optilab rEX online refractive index detector. 70 μL of each sample at 1 mg/mL was fractionated in GF150 buffer using a Superdex 200 5/150 analytical gel filtration column (GE Healthcare) run at 0.1 mL/min on an Agilent 1200 series LC system collecting UV at 280 nm before then passing through the light scattering and refractive index detectors in a standard SEC MALS format. Protein concentration was determined from the excess differential refractive index based on 0.186 ΔRI for 1 g/mL. The measured protein concentration and scattering intensity were used to calculate molecular mass from the intercept of a Debye plot using Zimm’s model as implemented in the Wyatt ASTRA software. The instrumental setup was verified using a 2 mg/mL BSA standard run of the same volume as experimental runs. The BSA monomer peak was used to check mass determination and to evaluate interdetector delay volumes and band broadening parameters that were then applied to experimental runs during analysis.

### Dynein motor domain pulldown

Equilibration buffer (GF150 buffer supplemented with 0.1 mM Mg-ATP and 10% glycerol) was used to equilibrate 20 μL of SNAP-Capture Magnetic Beads (New England Biolabs) slurry in 1.5 mL Protein Lo Bind Tubes (Eppendorf). SNAP-tagged LIS1 constructs were first covalently coupled to beads by incubating 50 μL of 1 μM of the respective protein at room temperature for 30 min. Beads were washed once with 1 mL of equilibration buffer and once with 1 mL binding buffer (GF150 supplemented with 0.05% IGEPAL (MilliporeSigma), 1 mM ATP, 1 mM Na_3_VO_4_ (New England Bioscience) and 10% glycerol). Beads with then incubated with 50 μL solution containing 0.2 μM dynein motor domain gently shaken for 1 hr at room temperature. The supernatant was removed and beads were washed twice with 500 uL binding buffer. 40 μL of SDS loading dye (ThermoFisher Scientific) was then added to the beads followed by incubation at 95°C for 5 min. 20 μL of this sample was analysed via SDS-PAGE and visualized by Coomassie Blue staining. The abundance of dynein motor was determined using densitometry in ImageJ. Three technical replicates were done per condition.

### Cell lines

HeLa GFP-BICD2N(1-400)-MTS and GFP-MTS cell lines were generated using the FLP-FRT recombination system. More specifically, Flp-In T-REX HeLa cells were transfected with 1.8 μg of pOGG4 plasmid and 200 ng of pcDNA5/FRT/TO plasmid encoding either a GFP-BICD2N-MTS or a GFP-MTS cassette using FuGENE HD (Promega). Cells with a stably integrated transgene were selected with 150 μg/mL hygromycin over 4-5 days. Cells were maintained in Dulbecco’s modified Eagle’s medium supplemented with 10% FBS and 1% penicillin/streptomycin. Transgene expression was induced by adding 10 μg/mL tetracycline to the culture medium.

### Mitochondrial relocation assay

HeLa GFP-BICD2N-MTS cells were transfected with 20 nM of either Non-Targeting siRNA #1 D-001810-01-05 (Dharmacon), ON-TARGETplus *PAFAH1B1* siRNA J-010330-07-0002 (Dharmacon) or SMARTPool: ON-TARGETplus *DYNC1H1* siRNA L-006828-00-0005 (Dharmacon) using Lipofectamine RNAiMax (ThermoFisher). Electroporations were performed 48 hours after siRNA transfection using the Neon Transfection System (ThermoFisher). Cells were resuspended in Resuspension Buffer R (ThermoFisher) at a density of 20x10^6^ cells/mL. 13 μL of cells were mixed with 2.5 μL of purified protein for a final protein concentration of 550 nM. Cells were pulsed twice at 1400 V for 2 ms using a Neon pipette tip and were immediately recovered in pre-warmed DMEM medium containing 10% FBS and 10 μg/mL tetracycline. Electroporated cells were plated on an imaging dish pre-coated with poly-D-lysine and incubated at 37°C for 8 hours to allow for cell spreading. Staining was performed with 20 μM Hoechst (ThermoFisher), 5 μg/mL CellMask Orange (ThermoFisher) and 50 nM MitoTracker Deep Red (ThermoFisher) for 20 minutes at 37°C. Samples were imaged using a Nikon Ti2 inverted fluorescence microscope with a 20x/0.75NA Air lens.

### Immunoblotting analysis and antibodies

For Western blot analysis, membranes were blocked with 5% milk in TBST for 1 hour at room temperature. Primary antibody incubation was carried out in 5% milk in TBST either for 2 hours at room temperature or for 14 hours at 4°C. Secondary antibody incubation was performed in 5% milk in TBST for 1 hour at room temperature. Primary antibodies used were: Mouse monoclonal anti-LIS1 (Cat no. sc-374586, Santa Cruz Biotechnology, 1:1000 dilution), Mouse monoclonal anti-Dynein IC1/2 (Cat no. sc-13524, Santa Cruz Biotechnology, 1:1000 dilution), Mouse monoclonal anti-GAPDH (Cat no. ab8245, Abcam, 1:1000 dilution), Rabbit polyclonal anti-SNAP-tag (Cat no. P9310S, New England Biolabs, 1:1000 dilution). Secondary antibodies used were: Goat anti-mouse IgG HRP (Cat no. P0447, Dako, 1:1500 dilution) and Goat anti-rabbit IgG HRP (Cat no. ab6721, Abcam, 1:2000 dilution).

### Quantification of mitochondrial spread

The spread of mitochondria relative to the nucleus was quantified using a custom ImageJ script (available at https://github.com/jboulanger/imagej-macro/tree/main/Cell_Organization) as previously described (115). The mitochondrial regions, the nucleus and the cell contour were defined by segmentation of MitoTracker, Hoechst and CellMask signals, respectively. The mitochondrial spread was measured as the trace of the second moment matrix of the MitoTracker signal intensity in each cell, akin to a standard deviation in 2D.

### Quantification and statistical analysis

Statistical analyses were performed with GraphPad Prism software or in R (116). The statistical details and specific statistical test used is mentioned in the respective figure caption. Plots were made with GraphPad Prism or in R

## Supplementary Material

Supplementary Material

Table S1

## Data Availability

Atomic coordinates and cryo-EM maps have been deposited in the Protein Data Bank (PDB) or Electron Microscopy Data Bank (EMDB), respectively, under accession codes 8PTK and 17873 (composite dynein-dynactin-JIP3-LIS1), 8PR2 and 17832 (dynein tail – JIP3 HBS1), 8PR3 and 17833 (dynein tail – JIP3 RH1 domain), 8PR4 and 17834 (pointed end–JIP3), 8PQY and 17828 (dynein-A motor domain + LIS1), 8PQW and 17826 (dynein-A motor domain-LIS1-CC1B-DIC-N), 8PQZ and 17829 (dynein-A1/A2 bound to LIS1), 8PQV and 17825 (dynein-B motor domain), 8PR0 and 17830 (dynein-A tail + IC-LC tower + p150), 8PR1 and 17831 (dynein-B tail + IC-LC tower), 17835 (consensus map of dynein-dynactin-JIP3^1-185^-LIS1), 17836 (consensus map of dynein-dynactin-JIP3^1-560^-LIS1) and 8PR5 (autoinhibited model of dynactin p150). All light microscopy data, AlphaFold2 models generated in this study, SDS-PAGE gels and Western blots are available at Zenodo (98).

## One Sentence Summary

Cryo-EM reveals a novel role of regulator LIS1 in forming active dynein-dynactin complexes.
