# Supplementary material for: Molecular mechanism of dynein-dynactin complex assembly by LIS1
Source: Science. Author manuscript; Available in PMC 2024 Apr 4. (PMC7615804; doi:10.1126/science.adk8544)
Supplement: Supplementary Material [file EMS194919-supplement-Supplementary_Material.docx]

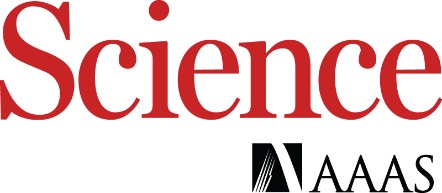


Supplementary Materials for

**Molecular mechanism of dynein-dynactin complex assembly by LIS1**

Kashish Singh, Clinton K. Lau, Giulia Manigrasso, José B. Gama, Reto Gassmann and Andrew P. Carter

Corresponding author: Andrew P. Carter, cartera@mrc-lmb.cam.ac.uk

**The PDF file includes:**

Figs. S1 to S10

Tables S2

References (117)

**Other Supplementary Materials for this manuscript include the following:**

Tables S1

Fig. S1.


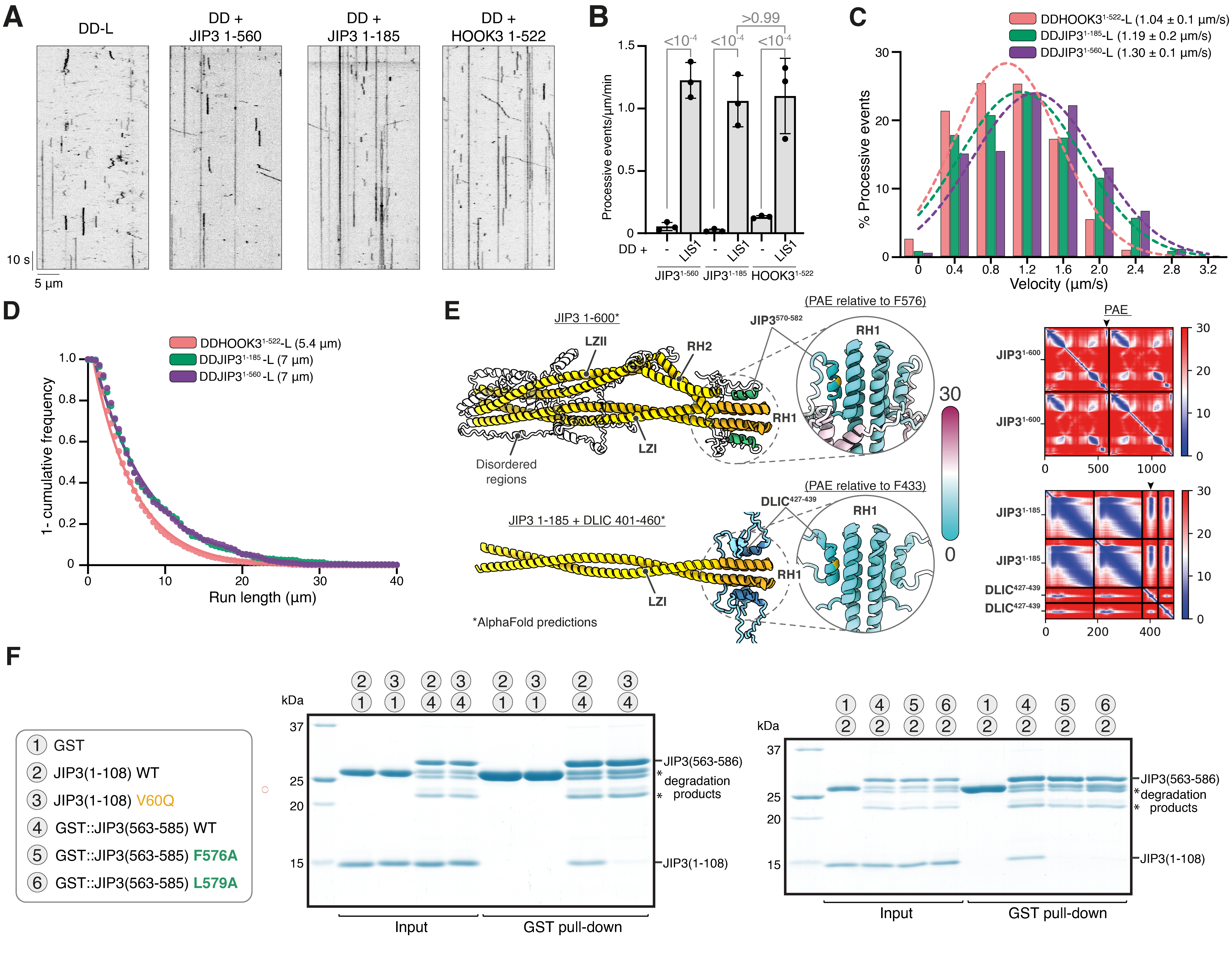


**Fig. S1. JIP3 is a dynein activating adaptor that undergoes autoinhibition. (A)** Kymographs of TMR-dynein-dynactin in presence of LIS1, JIP3^1-560^, JIP3^1-185^ and HOOK3^1-522^. **(B)** Quantification of the number of processive events per μm microtubule per minute with the mean ± S.D. plotted. The total number of movements analyzed were 26 (DDL), 74 (DD-JIP3^1-560^), 45 (DD-JIP3^1-185^), 224 (DD-HOOK3^1-522^). The data for DDJIP3^1-560^/JIP3^1-185^/HOOK3^1-522^-LIS1 is the same as displayed in Fig. 1C and is shown for comparison purposes. Experiments were performed with three technical replicates and statistical significance was determined using ANOVA with Tukey’s multiple comparison test. **(C)** Distribution of mean velocities of processive events. Gaussian fit of the distribution is shown as dotted lines along with mean velocity ± S.D. (n=3). **(D)** A 1-cumulative frequency distribution plot showing run length for dynein-dynactin-LIS1 with different adaptors fit to a one-phase exponential decay. The decay constant (run length) is shown. **(E)** AlphaFold2 prediction of two copies of JIP3 (1-600) (top) and JIP3 (1-185) along with two copies of DLIC2 (401-460) (bottom). The models in the zoom-in of the RH1 domains in the middle are colored based on PAE values (in Å) relative to that of the highlighted residues (yellow) where lower values represent higher confidence. The full PAE plot is shown on the right. **(F)** Coomassie Blue-stained SDS-PAGE gel of purified recombinant protein mixtures prior to the addition of glutathione agarose resin and of proteins eluted from glutathione agarose resin after GST pull-down. The gel on the left was displayed in Fig. 1D after cropping out the lanes for GST controls.

Fig. S2.

**
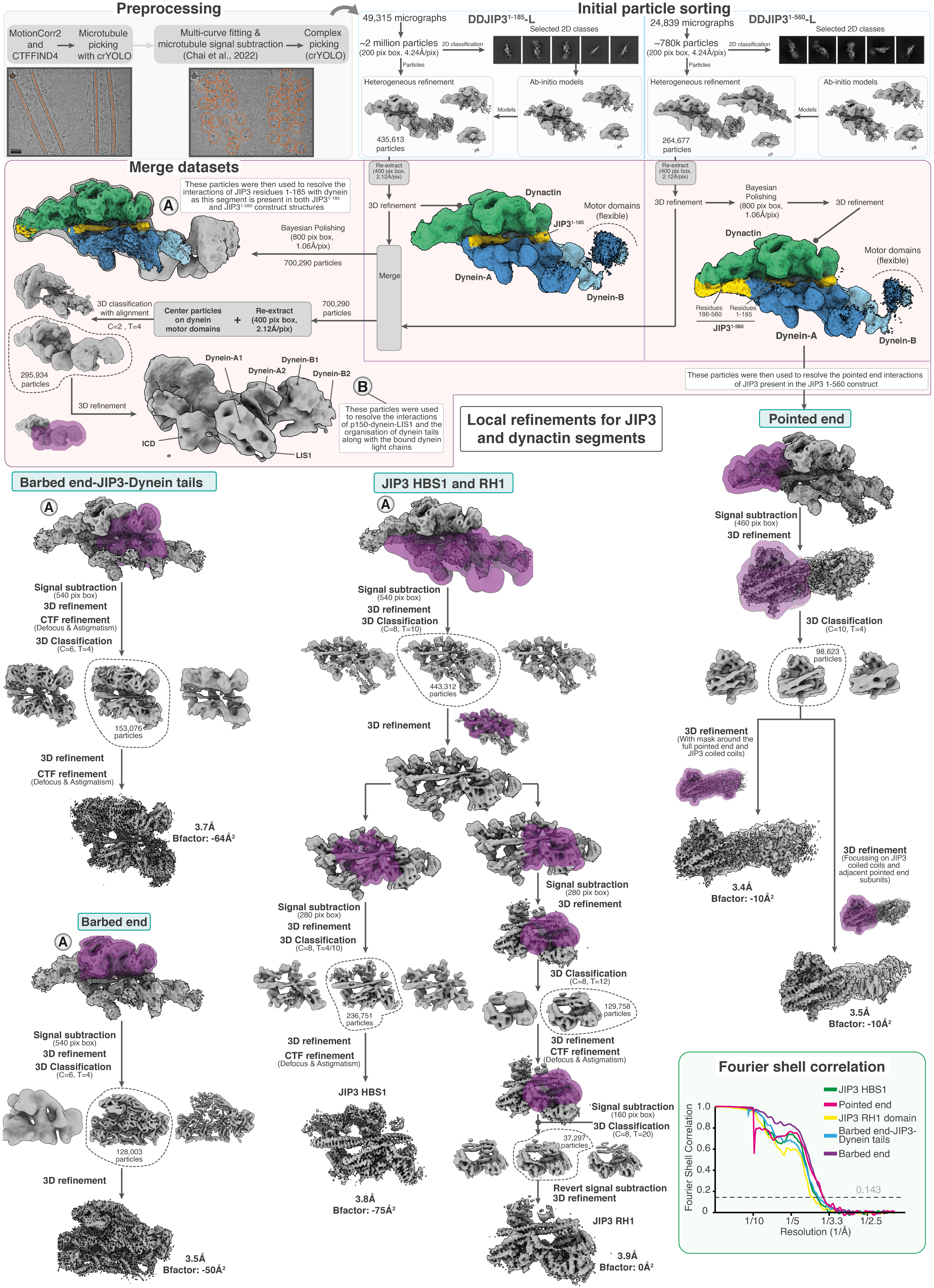
**


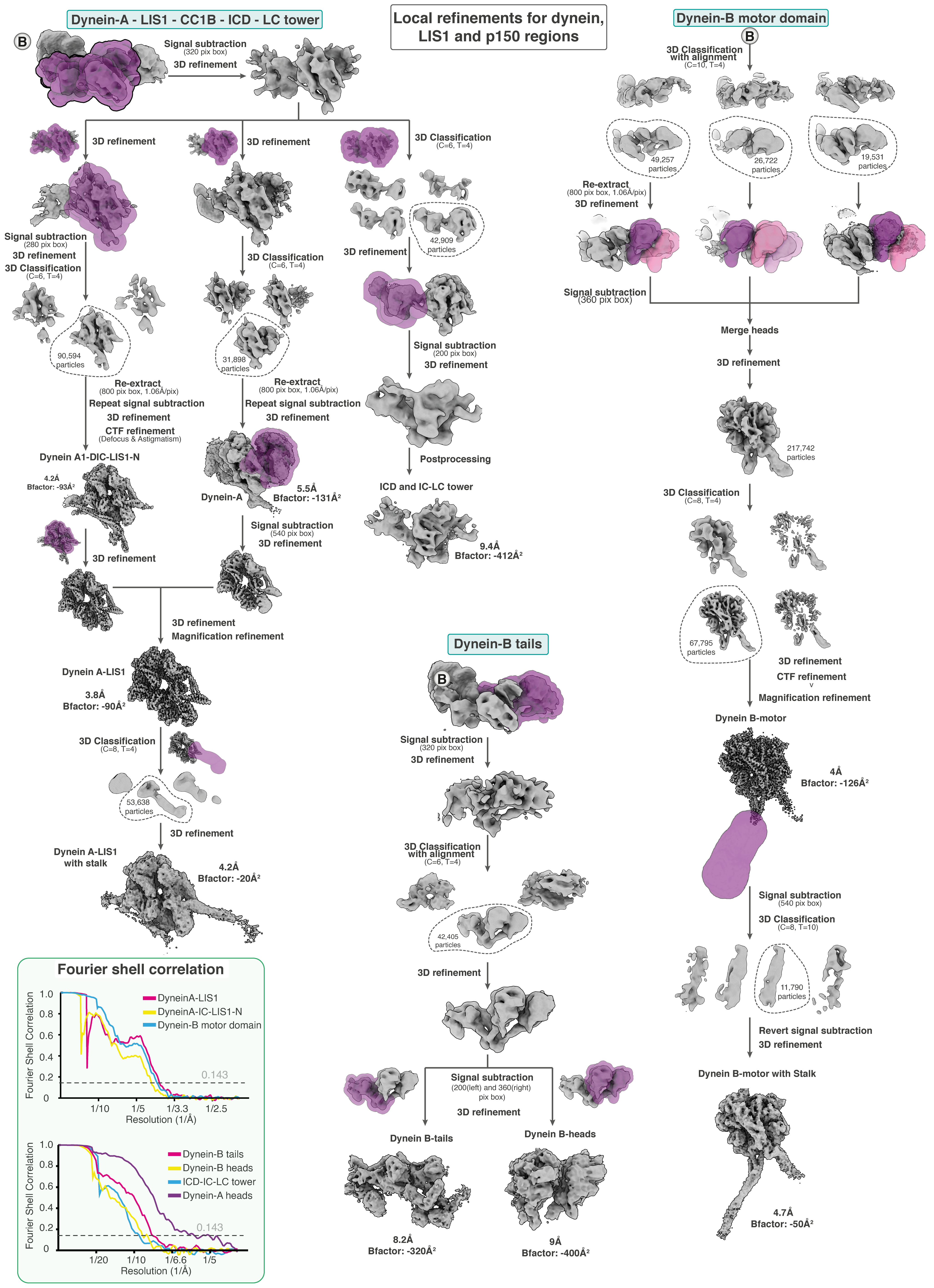


**Fig. S2. Cryo-EM image processing pipeline for dynein-dynactin-JIP3-LIS1 complexes.** (T = Tau fudge, C = number of classes). 3D classifications were performed without alignment unless otherwise specified. The classes selected after 3D classification are encircled using a dotted line. The masks used for signal subtraction, 3D classification and 3D refinements are displayed in purple. All defocus, magnification, and beam-tilt refinements were immediately followed by a 3D refinement (not shown). Plots show the gold standard Fourier shell correlation. The dotted horizontal line shows the 0.143 cut-off.


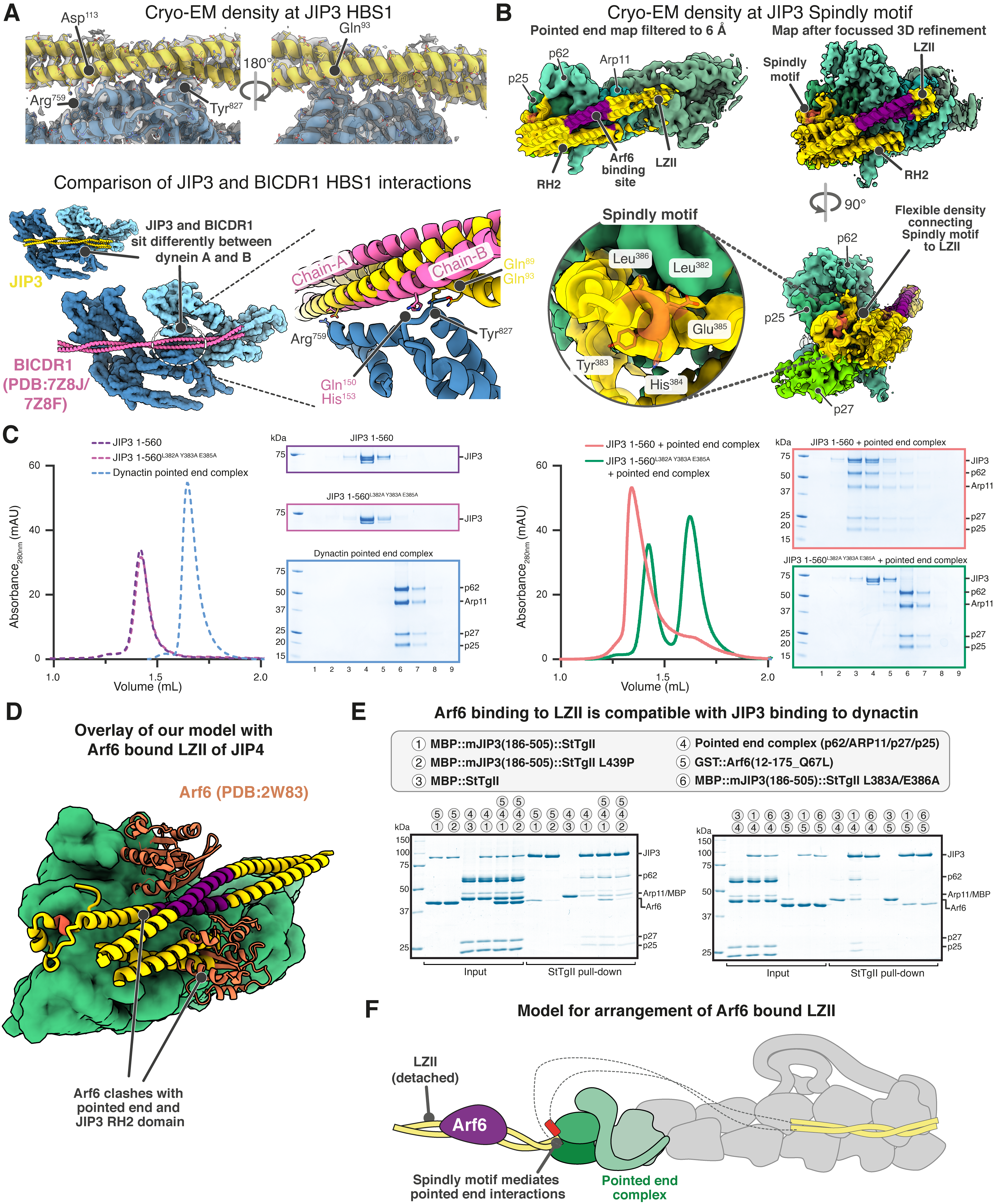
Fig. S3.

**Fig. S3. Interactions of JIP3 with dynein-dynactin.** (A) Cryo-EM density at the JIP3-HBS1 is shown (top). The trajectory of the JIP3 LZI and its interaction with dynein heavy chain are compared to those of BICDR1 (*27*). (B) Cryo-EM density of JIP3^1-560^ at the dynactin pointed end is shown. (C) Size-exclusion chromatography elution profiles from a Superose 6 3.2/300 increase column (left) and Coomassie Blue-stained SDS-PAGE gel (right) to compare complex formation between JIP3^1-560^ or JIP3^1-560^(L382A, Y383A, E385A) and pointed end complex. (D) Overlay of PDB-2W83 (Arf6 bound to JIP4-LZII) (*73*) with our model of JIP3 bound at the pointed end. (E) Coomassie Blue-stained SDS-PAGE gel of purified recombinant protein mixtures prior to the addition of Strep-Tactin Sepharose resin and of proteins eluted from the resin after strep-tag (StTgII) pull-down. (F) Schematic model for LZII orientation with respect to the pointed end when bound to Arf6.

Fig. S4.

**
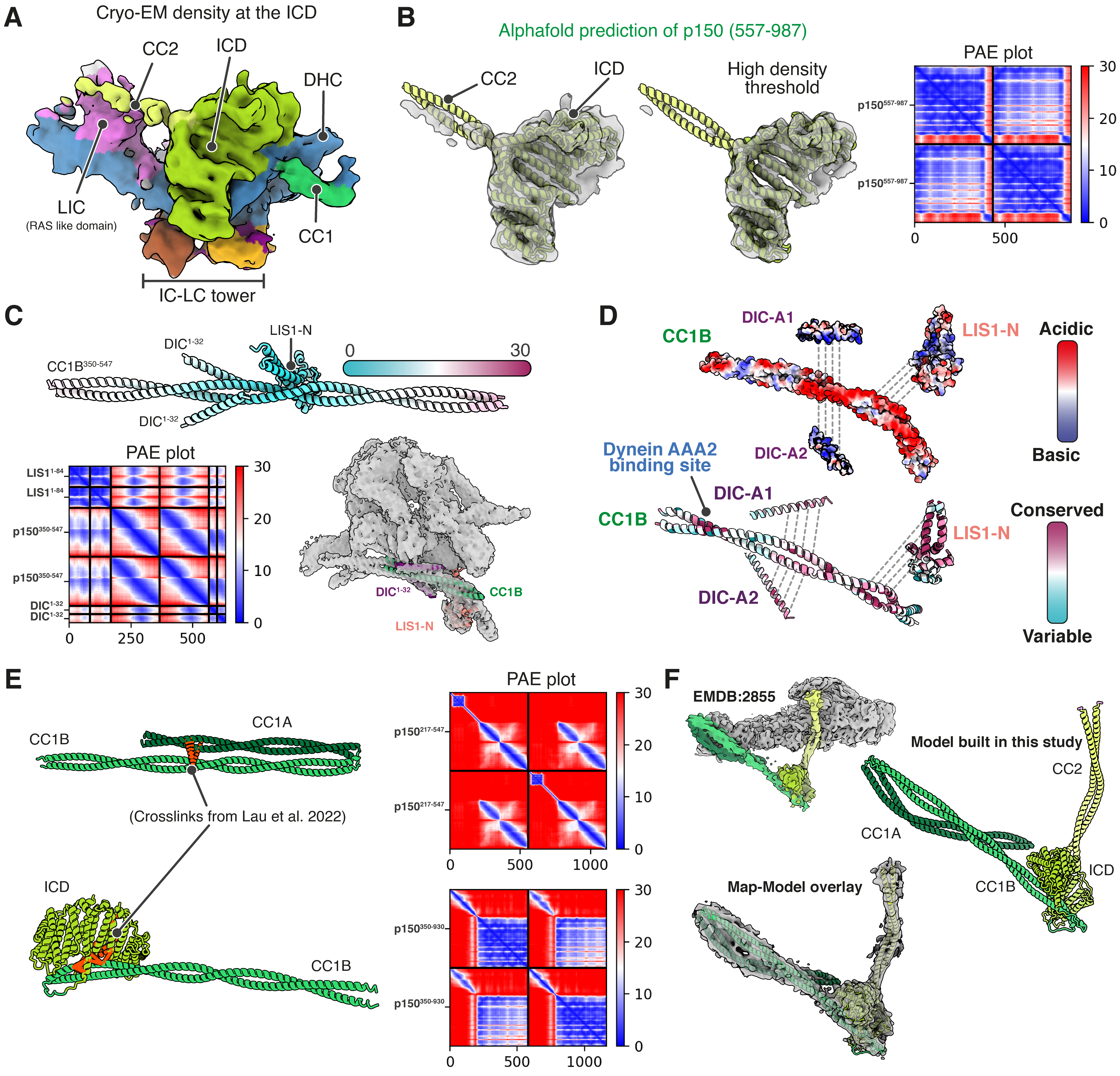
**

**Fig. S4. Modelling of dynactin p150 subunit and its bound DIC^1-32^ and LIS1-N. (A)** Cryo-EM map of ICD domain and IC-LC tower bound to dynein-A tail. **(B)** Fit of AlphaFold2 model of ICD and initial part of CC2 in the cryo-EM map at low threshold (left) and high threshold (middle). The PAE plot for the model is shown on the right. **(C)** AlphaFold2 prediction of DIC^1-32^ and LIS1-N binding to CC1B colored based on PAE values (in Å) relative to LIS1-K64 (top). The full PAE plot and the fit of this prediction in the experimental density are shown (bottom) **(D)** Electrostatic surface representation of CC1B-IC-LIS1-N to illustrate that their binding is predominantly driven by electrostatic interactions (top). Cartoon representation of CC1B, DIC^1-32^ and LIS1-N colored based on sequence conservation determined using the ConSurf web-server ([117](#biblioRef0116)) is depicted (bottom). **(E)** Arrangement of CC1A/B (top) and CC1B-ICD (bottom) predicted by AlphaFold2 is shown. All crosslinks between CC1A-CC1B generated by crosslinking dynactin with BS3 ([20](#biblioRef019)) are mapped on the model as red lines. PAE plots corresponding to the predictions are shown (right). **(F)** Cryo-EM structure of the autoinhibited form of dynactin where the different domains of the p150 subunit extending from the shoulder are highlighted (top). The model of the autoinhibited p150 built in this study is depicted in the middle and its fit into the cryo-EM density is shown at the bottom.

Fig. S5.

**
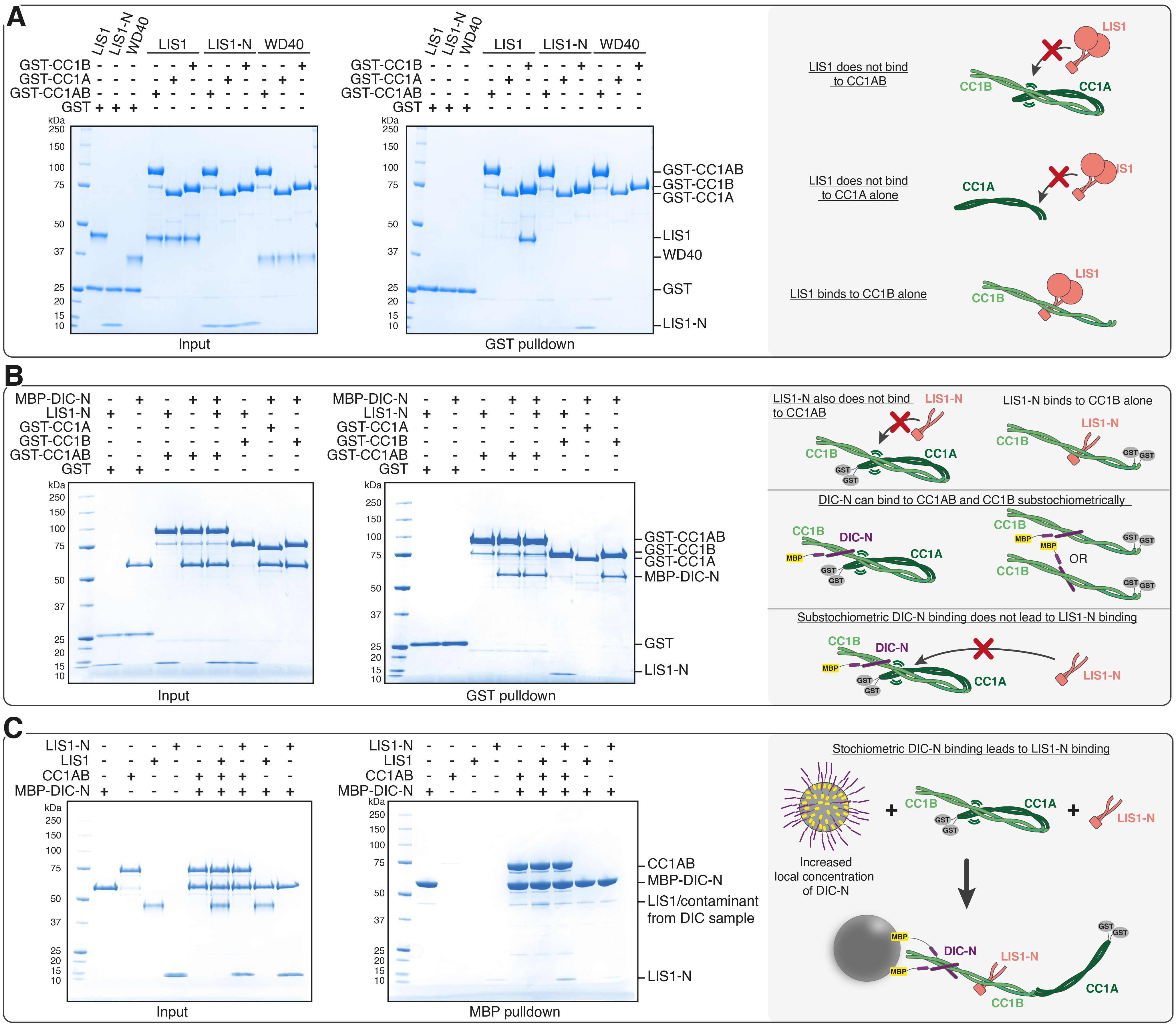
**

**Fig. S5. DIC-N helps overcome p150 autoinhibition (A), (B), (C)** Coomassie Blue-stained SDS-PAGE gels of purified recombinant protein mixtures prior to the addition of glutathione agarose or amylose resin and of proteins eluted from glutathione agarose or amylose resin after pull-down. Schematics summarizing the results are depicted next to each experiment.

Fig. S6.

**
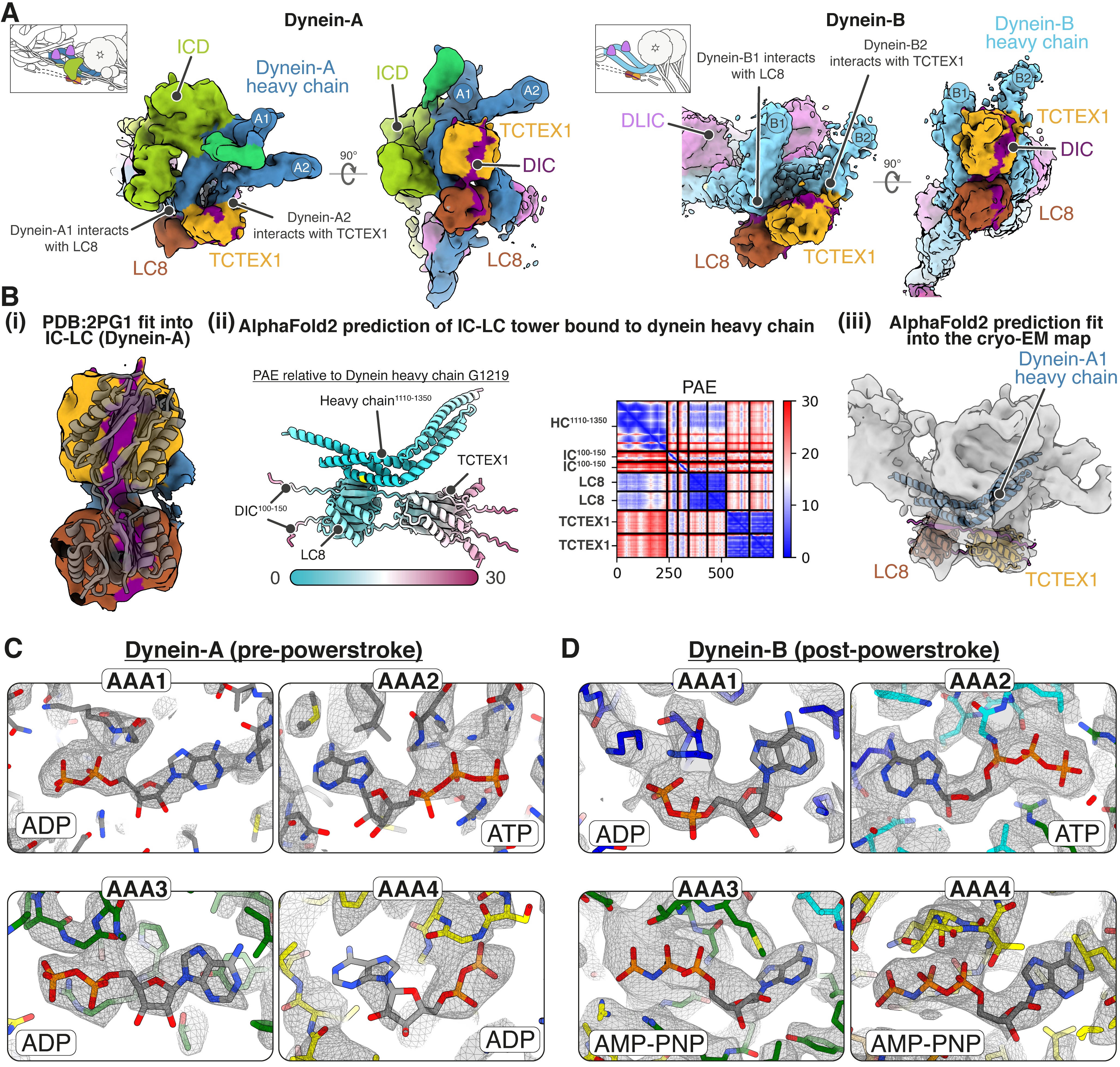
**

**Fig. S6. The IC-LC tower bridges the heavy chains of a dynein dimer. (A)** Cryo-EM maps of IC-LC tower bound to dynein-A (left) and dynein-B (right) heavy chains. **(B)(i)** Crystal structure of IC-LC tower (PDB-2PG1) ([68](#biblioRef067)) fit into the cryo-EM density of dynein-A IC-LC tower. **(ii)** AlphaFold2 model of dynein heavy chain with IC-LC tower colored based on the relative PAE (in Å) to dynein heavy chain residue G1219 and the full PAE plot are shown (right). The fit of this prediction into the cryo-EM density of dynein-A IC-LC tower is shown **(iii)**. **(C)** Model and density in the nucleotide pocket of AAA1-4 of dynein-A. **(D)** Model and density in the nucleotide pocket of AAA1-4 of dynein-B.

Fig. S7.


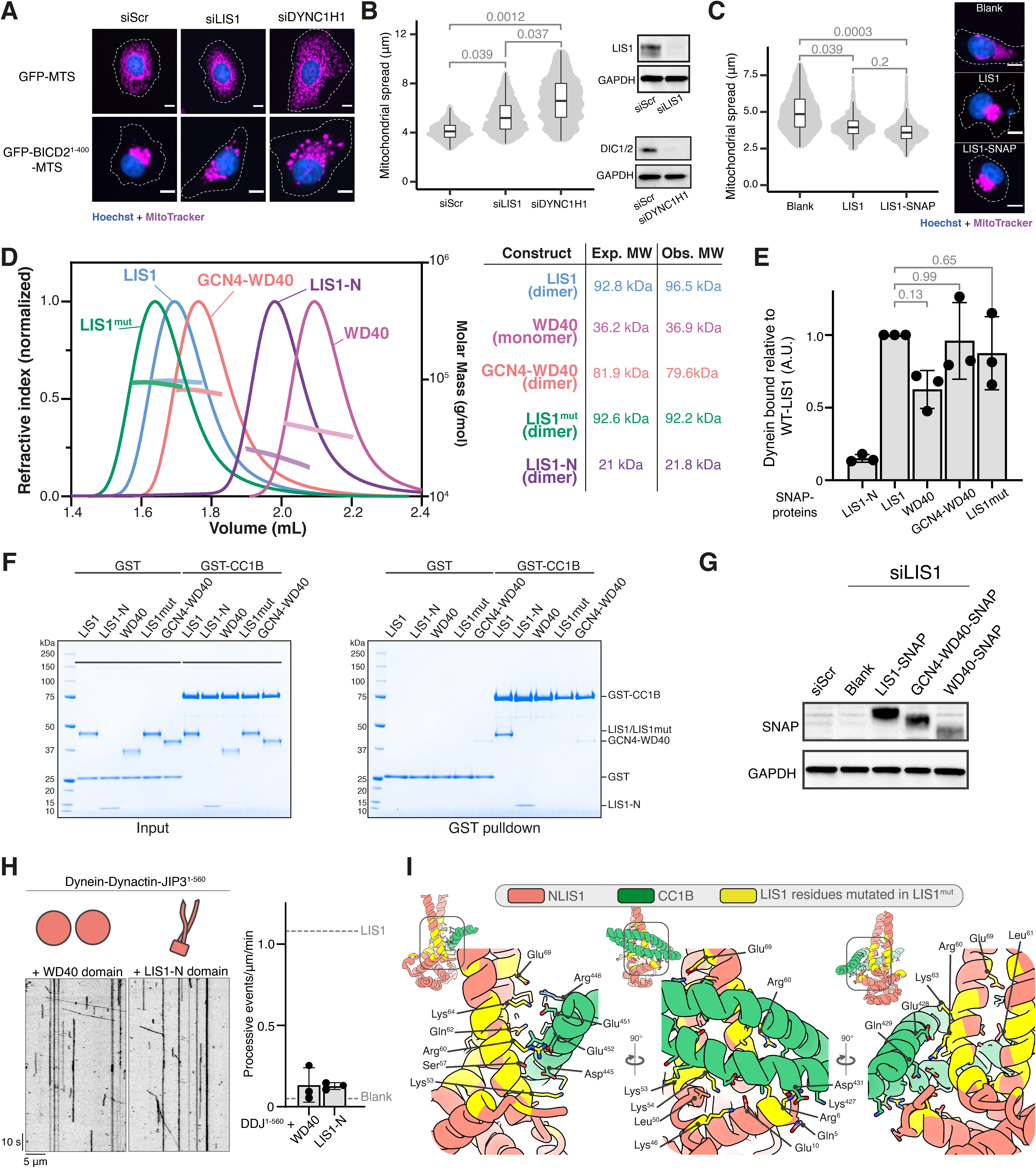


**Fig. S7. LIS1 requires both LIS1-N and WD40 domains to stimulate complex assembly. (A)** Representative images and **(B)** quantification showing the distribution of mitochondria (magenta) in HeLa GFP-BICD2N-MTS and GFP-MTS cells transfected with siRNAs against LIS1 and the dynein heavy chain (DYNC1H1). A scrambled siRNA was used as a negative control. Scale bars represent 10 μm. Data are plotted from 3 biological replicates. Knockdown efficiency was determined by western blot. Depletion of the dynein intermediate chain (DIC1/2) was used as a proxy for dynein heavy chain knockdown efficiency. **(C)** Comparison of LIS1 knockdown HeLa GFP-BICD2N-MTS cells rescued with either untagged LIS1 or LIS1-SNAP. Scale bars represent 10 μm. Data are plotted from 3 biological replicates. **(D)** SEC-MALS of purified LIS1 constructs. Mean observed molar mass (Obs.) and expected (Exp.) molar mass is indicated. **(E)** Quantification of pulldown of dynein motor domain by SNAP-tagged LIS1 constructs. The data is plotted from 3 technical replicates where the dynein binding was normalized using the LIS1-SNAP condition in each replicate. **(F)** Coomassie Blue-stained SDS-PAGE gel of purified recombinant protein mixtures prior to the addition of glutathione agarose resin and of proteins eluted from glutathione agarose resin after GST pull-down. **(G)** Transfection efficiency of SNAP-tagged LIS1 constructs used for rescuing LIS1 knockdown in Fig. 6 was determined by Western blot. **(H)** Kymographs and quantification of processive events per μm microtubule per minute of TMR-dynein-dynactin-JIP3^1-560^ in the presence of (from left) WD40 domains and LIS1-N. Cartoons depicting the LIS1 construct used are shown above each kymograph. Experiments were performed with three technical replicates**. (I)** Amino acids present in the interface between LIS1-N and CC1B. The amino acids colored in yellow were mutated to generate the LIS1^mut^ construct used in Fig. 6D and E. All statistical significance values were determined using ANOVA with Tukey’s multiple comparison.

Fig. S8.

**
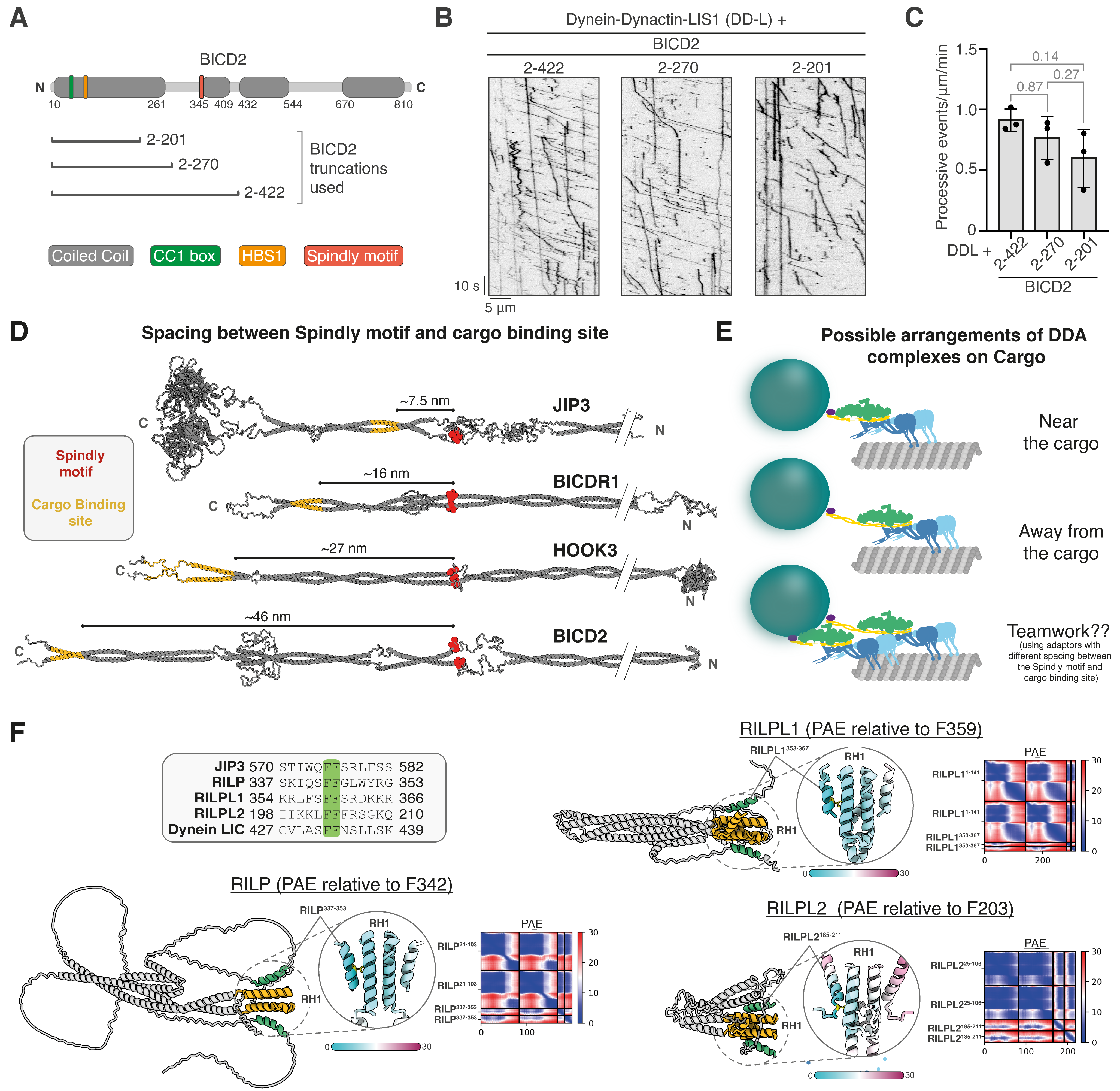
**

**Fig. S8. Dynein activation by BICD2 and conservation of inhibitory helices in members of the RH1 domain-containing proteins. (A)** Schematic representation of BICD2 adaptor and the length of the different constructs used in TIRF motility assays are depicted. **(B)** Kymographs of TMR-dynein-dynactin-LIS1 in the presence of different BICD2 constructs. Notably, the constructs 2-201 and 2-270 lack the spindly motif. **(C)** Quantification of the number of processive events/μm microtubule/minute with the mean ± S.D. plotted. The total number of events analyzed were 444 (BICD2^2-422^), 327 (BICD2^2-270^) and 327 (BICD2^2-201^). **(D)** Linearized AlphaFold2 models of the adaptors JIP3, BICDR1, HOOK3 and BICD2. The Spindly motif (red) and cargo binding regions (yellow) are mapped onto the model to show the approximate distance between the two sites. **(E)** Schematic showing possible arrangements of DDA complexes when recruited by different adaptors. **(F)** AlphaFold2 prediction of RH1 domain-containing proteins (RILP, RILPL1 and RILPL2). These proteins contain a C-terminally located helix containing phenylalanine residues (highlighted in green) that are predicted to bind the RH1 domain in a similar manner as JIP3.

Fig. S9.


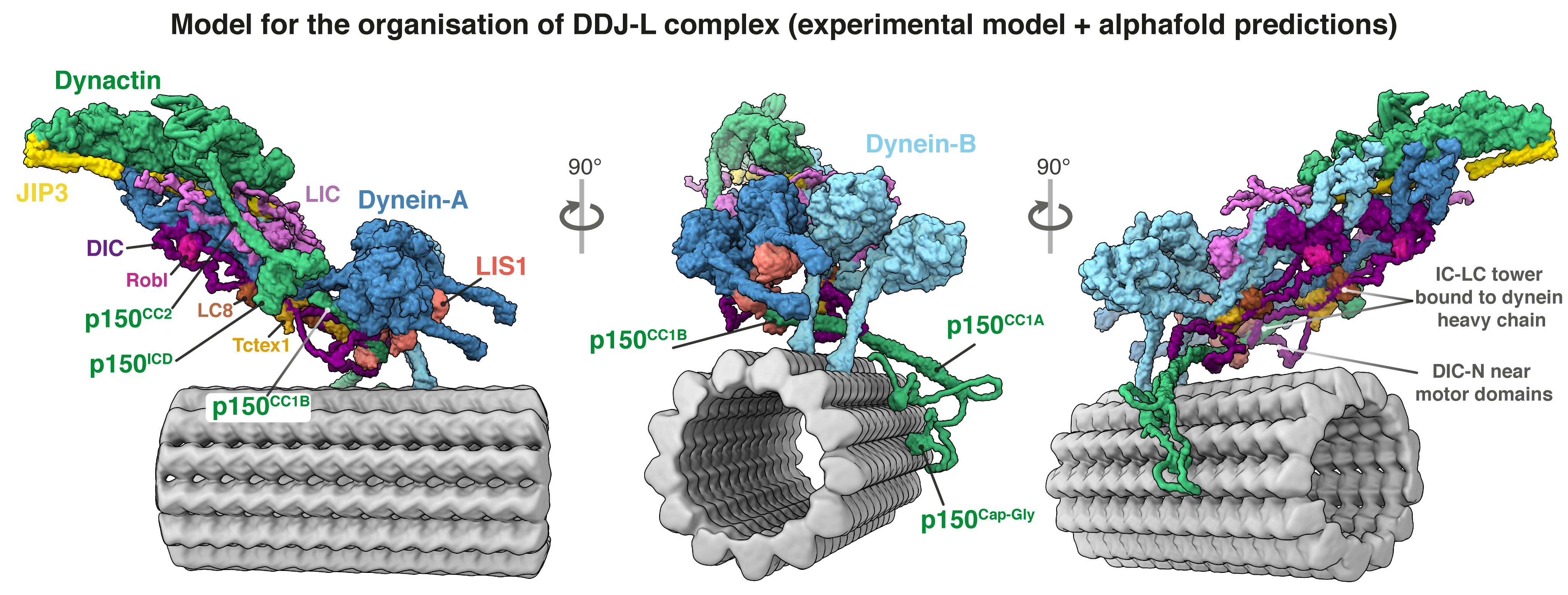


**Fig. S9. Model for organization of DDJ-L complex on microtubules.** Overall organization of the DDJ-LIS1 complex on microtubules is depicted. The N-terminal segments of p150 until CC1A and unstructured regions of DIC and DLIC were too flexible to be visualized in our cryo-EM structure and have been placed manually to illustrate where these segments are likely to be located in the complex.

Fig. S10.

**
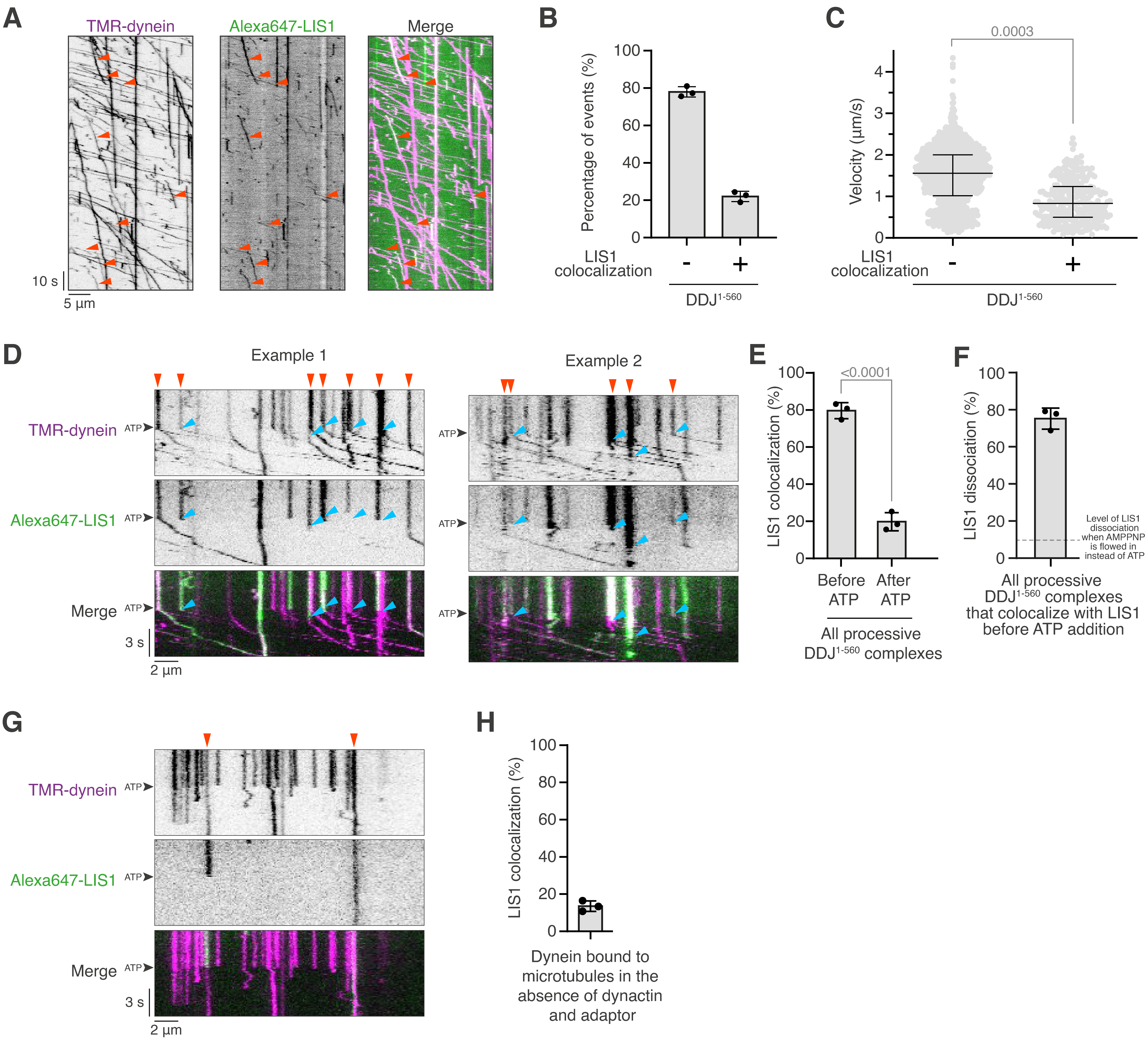
**

**Fig. S10. Colocalization of LIS1 with DDJ complexes. (A)** Kymographs of the TMR–dynein (left) and Alexa647–LIS1 (middle) channels and their overlay (right) when DDJ^1-560^-L complexes were imaged in TIRF motility assays. The orange arrowheads indicate the runs where LIS1 comigrates with the DDJ^1-560^ complex. **(B)** Quantification of processive events where Alexa-LIS1 does not and does co-migrate with TMR-dynein-dynactin-JIP3^1-560^ complexes. **(C)** Single-molecule velocity of TMR-dynein-dynactin-JIP3^1-560^ complexes in the absence or presence of co-migrating Alexa647-LIS1 with median ± interquartile range is plotted. The total number of events analyzed were 1022. The assay was performed with three technical replicates and statistical significance was determined with the median speeds from each replicate using an unpaired t test. **(D)** Kymographs of the TMR–dynein (top) and Alexa647–LIS1 (middle) channels and their overlay (bottom) when DDJ^1-560^-L complexes were pre-bound to microtubules using AMPPNP and then imaged while buffer containing ATP was flowed into the imaging chamber. Black arrow heads indicate when ATP enters the imaging chamber. Orange arrowheads indicate the processive DDJ^1-560^ complexes that colocalize with Alexa647-LIS1. Blue arrowheads indicate the processive DDJ^1-560^ where Alexa647-LIS1 dissociates upon initiation of movement of the complex. **(E)** Quantification of Alexa647-LIS1 colocalizing with processive DDJ^1-560^ complexes before and after ATP was flowed into the imaging chamber (80% colocalization before ATP and 20% after ATP). The assay was performed with three technical replicates and statistical significance was determined using an unpaired t test. **(F)** Quantification showing the amount of Alexa647-LIS1 dissociation (~75%) upon ATP addition from processive DDJ^1-560^ complexes that initially colocalized with LIS1. The dotted line indicates the background level of dissociation (~9.5%) that occurred due to the experimental setup in a control sample where AMPPNP was flowed into the imaging chamber instead of ATP. **(G)** Kymographs of the TMR–dynein (top) and Alexa647–LIS1 (middle) channels and their overlay (bottom) when TMR-dynein was pre-bound to microtubules (in the absence of dynactin and activating adaptor) using AMPPNP and then imaged while buffer containing ATP was flowed into the imaging chamber. Black arrow heads indicate when ATP enters the imaging chamber. Orange arrowheads indicate the dynein molecules that colocalize with Alexa647-LIS1. **(H)** Quantification of Alexa647-LIS1 colocalizing with dynein molecules before ATP was flowed into the imaging chamber (13.5% colocalization). The assay was performed with three technical replicates. The low colocalization of LIS1 with dynein in the absence of dynactin and adaptor suggests that the high level of colocalization seen in the DDJ^1-560^ complexes (Panel D and E) are specific to dynein incorporated into a DDA complex.

**Table S1.** Quantification and statistics of in vitro and cellular assays.

T**able S2.** Cryo-EM data collection and refinement statistics

|  | **JIP3-HBS1** | | **JIP3-RH1** | | **Pointed end with JIP3 spindly motif** | | **Dynein-A motor domain/LIS1** | | **Dynein-A1 motor domain/LIS1/CC1B** | |
| --- | --- | --- | --- | --- | --- | --- | --- | --- | --- | --- |
| **EMDB ID** | 17832 | | 17833 | | 17834 | | 17828 | | 17826 | |
| **PDB ID** | 8PR2 | | 8PR3 | | 8PR4 | | 8PQY | | 8PQW | |
| **Data collection and processing** |  | |  | |  | |  | |  | |
| Magnification | 81000X | | 81000X | | 81000X | | 81000X | | 81000X | |
| Voltage | 300 | | 300 | | 300 | | 300 | | 300 | |
| Pixel size (Å) | 1.059 | | 1.059 | | 1.059 | | 1.059 | | 1.059 | |
| Electron exposure (e^−^/ Å^2^) | 54 | | 54 | | 54 | | 54 | | 54 | |
| Defocus range (μm) | 0.6-3.4 | | 0.6-3.4 | | 0.6-3.4 | | 0.6-3.4 | | 0.6-3.4 | |
| Initial particle images | 700290 | | 700290 | | 264677 | | 700290 | | 700290 | |
| Final particle images | 236751 | | 37297 | | 98623 | | 90594 | | 90594 | |
| Symmetry imposed | C1 | | C1 | | C1 | | C1 | | C1 | |
| Map Resolution (Å) | 3.8 | | 3.9 | | 3.5 | | 3.8 | | 4.2 | |
| FSC threshold | 0.143 | | 0.143 | | 0.143 | | 0.143 | | 0.143 | |
| Sharpening B factor (Å^2^) | -75 | | - | | -10 | | -90 | | -93 | |
| **Model Refinement** |  | |  | |  | |  | |  | |
| Model Resolution (Å) | 4 | | 4.1 | | 3.9 | | 4.1 | | 4.4 | |
| FSC threshold | 0.5 | | 0.5 | | 0.5 | | 0.5 | | 0.5 | |
| **Model composition** |  | |  | |  | |  | |  | |
| Non-hydrogen atoms | 14913 | | 18046 | | 9494 | | 27918 | | 30493 | |
| Protein residues | 1816 | | 2239 | | 1322 | | 3518 | | 4035 | |
| Ligands | - | | - | | ZN:3, ATP:1 | | MG:2, ADP:3, ATP:1 | | MG:2, ADP:3, ATP:1 | |
| **Mean B factors (Å^2^)** |  | |  | |  | |  | |  | |
| Protein | 94 | | 145 | | 129 | | 116 | | 141 | |
| Ligand | - | | - | | 110 | | 94 | | 110 | |
| **RMSD deviations** |  | |  | |  | |  | |  | |
| Bond lengths (Å) | 0.005 | | 0.006 | | 0.008 | | 0.004 | | 0.004 | |
| Bond angles (°) | 1.04 | | 1.08 | | 1.33 | | 0.891 | | 0.86 | |
| **Validation** |  | |  | |  | |  | |  | |
| Clashscore | 4.9 | | 5.91 | | 4.09 | | 5.5 | | 5.96 | |
| MolProbity score | 1.34 | | 1.5 | | 1.66 | | 1.56 | | 1.55 | |
| Rotamers outliers (%) | 0.62 | | 0.66 | | 1 | | 0.3 | | - | |
| **Ramachandran plot** |  | |  | |  | |  | |  | |
| Favored (%) | 97.6 | | 96.96 | | 92.43 | | 96.18 | | 96.54 | |
| Allowed (%) | 2.4 | | 2.99 | | 7.5 | | 3.79 | | 3.46 | |
| Outliers (%) | - | | 0.05 | | 0.08 | | 0.03 | | - | |
|  | | **Dynein-A heavy chain/IC-LC tower/ICD** | | **Dynein-B motor domain** | | **Dynein-B heavy chain/IC-LC tower** | | **Dynein-A1-A2/ LIS1** | |  |
| **EMDB ID** | | 17830 | | 17825 | | 17831 | | 17829 | |  |
| **PDB ID** | | 8PR0 | | 8PQV | | 8PR1 | | 8PQZ | |  |
| **Data collection and processing** | |  | |  | |  | |  | |  |
| Magnification | | 81000X | | 81000X | | 81000X | | 81000X | |  |
| Voltage | | 300 | | 300 | | 300 | | 300 | |  |
| Pixel size (Å) | | 1.059 | | 1.059 | | 1.059 | | 1.059 | |  |
| Electron exposure (e^−^/ Å^2^) | | 54 | | 54 | | 54 | | 54 | |  |
| Defocus range (μm) | | 0.6-3.4 | | 0.6-3.4 | | 0.6-3.4 | | 0.6-3.4 | |  |
| Initial particle images | | 700290 | | 700290 | | 700290 | | 700290 | |  |
| Final particle images | | 42909 | | 67795 | | 42405 | | 31898 | |  |
| Symmetry imposed | | C1 | | C1 | | C1 | | C1 | |  |
| Map Resolution (Å) | | 9.4 | | 4 | | 8.2 | | 5.5 | |  |
| FSC threshold | | 0.143 | | 0.143 | | 0.143 | | 0.143 | |  |
| Sharpening B factor (Å^2^) | | -412 | | -126 | | -320 | | -131 | |  |
| **Model Refinement** | |  | |  | |  | |  | |  |
| Model Resolution (Å) | | 10.6 | | 4 | | 8.8 | | 7.2 | |  |
| FSC threshold | | 0.5 | | 0.5 | | 0.5 | | 0.5 | |  |
| **Model composition** | |  | |  | |  | |  | |  |
| Non-hydrogen atoms | | 13123 | | 24537 | | 17872 | | 37643 | |  |
| Protein residues | | 2649 | | 3032 | | 3611 | | 7553 | |  |
| Ligands | | - | | ANP:2, ADP:2, ATP:1 | | - | | MG:4, ADP:6, ATP:2 | |  |
| **Mean B factors (Å^2^)** | |  | |  | |  | |  | |  |
| Protein | | 786 | | 108 | | 375 | | 226 | |  |
| Ligand | | - | | 89 | | - | | 184 | |  |
| **RMSD deviations** | |  | |  | |  | |  | |  |
| Bond lengths (Å) | | 0.007 | | 0.005 | | 0.007 | | 0.005 | |  |
| Bond angles (°) | | 1.45 | | 0.888 | | 1.45 | | 1.191 | |  |
| **Validation** | |  | |  | |  | |  | |  |
| Clashscore | | 1.47 | | 6.01 | | 1.16 | | 0.7 | |  |
| MolProbity score | | 1.06 | | 1.52 | | 1.13 | | 0.95 | |  |
| Rotamers outliers (%) | | - | | 0.52 | | - | | - | |  |
| **Ramachandran plot** | |  | |  | |  | |  | |  |
| Favored (%) | | 96.05 | | 96.85 | | 95.6 | | 96.53 | |  |
| Allowed (%) | | 3.05 | | 3.05 | | 4.34 | | 3.45 | |  |
| Outliers (%) | | - | | 0.1 | | 0.06 | | 0.01 | |  |

|  | **Composite map of DDJL** | **DDJ1-185 Consensus map** | **DDJ1-560 consensus map** | **Autoinhibited Dynactin p150** |
| --- | --- | --- | --- | --- |
| **EMDB ID** | 17873 | 17835 | 17836 | 2855 (Urnavicius et al. 2015) |
| **PDB ID** | 8PTK | - | - | 8PR5 |
| **Data collection and processing** |  |  |  |  |
| Magnification | 81000X | 81000X | 81000X | - |
| Voltage | 300 | 300 | 300 | - |
| Pixel size (Å) | 1.059 | 1.059 | 1.059 | - |
| Electron exposure (e^−^/ Å^2^) | 54 | 54 | 54 | - |
| Defocus range (μm) | 0.6-3.4 | 0.6-3.4 | 0.6-3.4 | - |
| Initial particle images | - | 435613 | 264677 | - |
| Final particle images | - | 435613 | 264677 | - |
| Symmetry imposed | C1 | C1 | C1 | - |
| Map Resolution (Å) | Filtered to 10Å | 4.5 | 4.9 | - |
| FSC threshold | - | 0.143 | 0.143 | - |
| Sharpening B factor (Å^2^) | - | - | - | - |
| **Model Refinement** |  |  |  |  |
| Model Resolution (Å) | - | - | - | 13.8 |
| FSC threshold | - | - | - | 0.5 |
| **Model composition** |  |  |  |  |
| Non-hydrogen atoms | 161773 | - | - | 8326 |
| Protein residues | 32518 | - | - | 1676 |
| Ligands | ATP:6, ADP:16, ANP:4, ZN:3, MG:6 | - | - | - |
| **Mean B factors (Å^2^)** |  |  |  |  |
| Protein | 378 | - | - | 502 |
| Ligand | 152 | - | - | - |
| **RMSD deviations** |  |  |  |  |
| Bond lengths (Å) | 0.006 | - | - | 0.006 |
| Bond angles (°) | 1.342 | - | - | 1.451 |
| **Validation** |  |  |  |  |
| Clashscore | 0.59 | - | - | 3.43 |
| MolProbity score | 0.94 | - | - | 1.21 |
| Rotamers outliers (%) | - | - | - | - |
| **Ramachandran plot** |  |  |  |  |
| Favored (%) | 96.39 | - | - | 97.66 |
| Allowed (%) | 3.56 | - | - | 2.34 |
| Outliers (%) | 0.04 | - | - | - |
